# Global burden of childhood otitis media attributable to secondhand smoke from 1990 to 2021: a systematic analysis of the global burden of disease study 2021

**DOI:** 10.3389/fped.2025.1619721

**Published:** 2025-12-01

**Authors:** Bei Li, Qingchun Pan, Kai Zou

**Affiliations:** 1 Department of Otolayngology, Head and Neck Surgery, Affliated Hospital of NorthSichuan Medical College, Nanchong, China; 2 School of Lmaging, North Sichuan Medical College, Nanchong, China

**Keywords:** childhood otitis media, secondhand smoke, joinpoint regression analysis, health inequality analysis, autoregressive integrated moving average, global burden of disease 2021

## Abstract

**Background:**

Childhood otitis media is significantly influenced by exposure to secondhand smoke (SHS), posing a considerable challenge to pediatric health. Utilizing data obtained from the Global Burden of Disease Study (GBD) 2021, this population-based burden analysis examines the worldwide impact of SHS-associated otitis media among children aged 0–14 years between 1990 and 2021, while forecasting epidemiological patterns up to 2051.

**Methods:**

The GBD 2021 dataset served as the foundation for evaluating four key metrics: disability-adjusted life years (DALYs), age-standardized rates for DALYs (ASDR), years lived with disability (YLDs), and age-standardized YLD rates (ASYR) associated with pediatric middle ear infections caused by SHS exposure. Joinpoint regression modeling was employed to detect epidemiological trend change points, while disparity assessment in health outcomes examined variations across socioeconomic and demographic strata. Burden projection was conducted through implementation of an autoregressive integrated moving average (ARIMA) statistical framework.

**Results:**

The global YLDs due to childhood otitis media attributable to SHS decreased by 17.65% in 1990–2021. The ASYR decreased by 28.78%, from 5.49 [95% uncertainty interval (UI): 2.38–9.9] per 100,000 to 3.91 (95% UI: 1.66–7.23) per 100,000. The ASDR falling by 32.08%, from 5.83 (95% UI: 2.61–10.37) per 100,000 to 3.96 (95% UI: 1.68–7.31) per 100,000. The burden was consistently greater in males compared to females, with the highest impact observed in regions with low and middle SDI regions. Joinpoint regression analysis revealed the greatest decline in ASYR occurred in 2003–2008, while the most significant decrease in ASDR occurred in 1994–1999. Health inequality analysis showed a reduction in inequality from 1990 to 2021. ARIMA projections suggest a further decline in global ASYR and ASDR by 2051, although an increase may occur in middle SDI regions.

**Conclusion:**

While there has been a general decrease in the global burden of childhood otitis media linked to secondhand smoke exposure. Certain regions (notably Central Asia, North Africa/Middle East, and Oceania) continue to experience a disproportionately high burden. These findings call for continued investment in public health strategies that not only limit SHS exposure but also address healthcare access gaps in underserved populations.

## Introduction

Otitis media represents inflammatory pathology affecting the middle ear cavity located medial to the tympanic membrane. This anatomical compartment establishes connection with the upper respiratory system through the eustachian tube, which under physiological conditions maintains pressure equilibrium and facilitates ventilation (1). Recent population-based studies in Turkey have quantified significant socioeconomic gradients in SHS exposure, demonstrating that women with lower educational attainment and income levels experience disproportionately higher exposure risks (2), while older adult populations show distinct vulnerability patterns (3). These findings underscore the need for population-specific assessments of SHS health impacts. Under normal conditions, this tube regulates air pressure and ventilation. Infants and young children are particularly susceptible to otitis media due to their immature Eustachian tube anatomy - characterized by shorter length, horizontal orientation, and poorer muscular function (4). However, when it becomes blocked—due to infections, allergic reactions, or structural abnormalities such as adenoid hypertrophy, cleft palate, or craniofacial malformations—the resulting negative pressure and fluid buildup can facilitate microbial growth, leading to otitis media (5). This condition, especially common in young children (typically aged 0–5 years), remains a leading cause of preventable hearing loss worldwide. Estimates suggest it may account for nearly half of all pediatric hearing loss case (6, 7). Frequently arising from upper respiratory infections, acute otitis media demonstrates recurrent characteristics that impose significant strain on both healthcare infrastructure and family units (8, 9). Detailed comprehension of pediatric otitis media epidemiology provides essential insights for establishing robust preventive frameworks and advancing timely therapeutic interventions.

The etiology of otitis media is multifactorial, involving immunological, anatomical, genetic, and environmental components. Among the modifiable risk factors, exposure to secondhand smoke (SHS) has been consistently linked to increased susceptibility (10). SHS introduces harmful substances, such as nicotine and other irritants, which compromise the respiratory and immune defense systems in children (11). Tobacco smoke contains aldehydes, nicotine, and particulate matter that damage ciliary motility, induce mucosal inflammation, and impair mucociliary clearance in the eustachian tube, leading to negative middle ear pressure and fluid accumulation (12). Data indicate that a significant proportion of children with acute otitis media (AOM), up to 78%, are regularly exposed to SHS, with household surveys showing that 76.5% of families with children report smoking occurring in their presence, yet public awareness of this risk remains low (13, 14).

The Global Burden of Disease (GBD) 2021 study offers a robust dataset to quantify the health impact of SHS on childhood otitis media at global and regional levels. Leveraging this dataset, the present study aims to provide a comprehensive assessment of the burden of otitis media attributable to SHS. Specifically, we: (1) describe the global distribution and burden of otitis media linked to SHS; (2) examine temporal trends at global, regional, and national levels; (3) apply Joinpoint regression to evaluate trend changes; (4) assess cross-national inequality using World Health Organization (WHO)-recommended equity metrics; and (5) project the burden through 2051. These findings aim to inform public health actions, support policy development, and contribute to global efforts to reduce preventable childhood illnesses.

## Methods

### Data source

The research employed epidemiological records sourced from the GBD 2021 repository developed by IHME. Encompassing 204 geopolitical regions, this dataset delivers systematic evaluations of health loss attributable to 371 pathological conditions and traumatic events, supplemented with 88 contributory risk factor analyses spanning 1990–2021 (15). Data acquisition involved retrieving pediatric otitis media cases linked to SHS exposure in 0–14 year-olds through the Global Health Data Exchange interface. Through standardized nosological classifications and unrestricted availability of worldwide epidemiological metrics, this platform enables comparative assessments. Ethical clearance requirements were waived given the exclusively open-access nature of the information repositories involved.

### Data collection

Epidemiological metrics including disability-adjusted life years (DALYs), morbidity-associated healthy life years (YLDs), age-adjusted DALY rates (ASDR), and corresponding YLD metrics (ASYR) were extracted from the GBD 2021 repository. DALYs quantify disease burden through aggregation of premature mortality-derived life years and morbidity-related health deterioration periods. YLDs denote healthy life years compromised by pathological or traumatic conditions, principally characterizing population-level quality-of-life deterioration attributable to disease processes. In line with the GBD framework, we quantified the population health impact using metrics of disease burden (DALYs and YLDs) instead of incident case counts. It should be noted that this precludes the estimation of attributable cases, although it provides a comprehensive assessment of the overall burden.

### Definition

In the GBD 2021, otitis media cases were identified as middle ear infections meeting the ICD-10 diagnostic criteria (codes H65-H75.83) and were subsequently incorporated into the disease modeling framework. A limitation of the GBD framework is that the otitis media case definition encompasses acute, chronic, and effusive forms without differentiation. Therefore, our estimates represent the burden attributable to SHS across the entire spectrum of otitis media conditions, and we cannot isolate the effect on acute episodes alone.

### Socio-demographic index (SDI)

SDI represents a composite index derived from three socioeconomic indicators: per capita income levels, educational achievement metrics, and fertility rate statistics. This methodological framework establishes a comparative developmental ranking system (0–1 scale) for national evaluation (16). Socioeconomic development quintiles were stratified into five tiers: low, low-middle, middle, high-middle, and high (17). Analytical procedures examined correlations between middle ear disease burden associated with SHS exposure and national development quintile classifications.

### Joinpoint regression analysis

To evaluate time-dependent changes in disease burden, we applied the Joinpoint regression model, it is a widely adopted tool for identifying and quantifying temporal trends. This method was used to examine variations in ASYR and ASDR for childhood otitis media attributable to SHS exposure (18).

### Health inequality analysis

Health disparity assessment employed two established metrics: the slope index of inequality (SII) and CI. SII computation involved weighted linear regression analysis where national-level DALY and YLD rates were modeled against respective SDI ranking positions. SDI continuum construction incorporated cumulative populationmidpoints stratified by development quintiles. Conversely, CI derivation required geometric integration below the Lorenz curve, aligning accumulated health metric values (YLDs/DALYs) with SDI-ordered population distributions. This graphical tool visualizes health outcome distribution patterns across socioeconomic gradients, whereas the CI numerically expresses disparity levels compared to equitable allocation scenarios (19).

### Predictive analysis

Longitudinal burden forecasting was conducted using the ARIMA (autoregressive integrated moving average) statistical framework. This forecasting technique systematically combines autoregressive components (AR), differencing operations (I), and moving average elements (MA) in time series analysis. In the ARIMA(p,d,q) parametrization, the notation “p” denotes autoregressive term quantity, “d” specifies differencing steps required for achieving stationarity, and “q” determines moving average component count. Stationarity was confirmed using Augmented Dickey-Fuller (*p* < 0.01) and KPSS (*p* > 0.05) tests. Parameter optimization and model configuration were implemented through the auto.arima algorithm within R statistical software (version 4.4.1). Residuals were tested for white noise (*p* > 0.05) to validate model fit. The validated predictive model generated 30-year projections for pediatric otitis media burden attributable to SHS exposure (20).

### Statistical analysis

Principal evaluation parameters comprised age-standardized DALYs and YLDs rates, alongside their normalized counterparts (ASDR and ASYR), all normalized per 100,000 demographic units following GBD protocols. These metrics were presented alongside 95% confidence intervals (UI) in accordance with established methodologies. Temporal burden patterns were assessed through annualized percentage change estimations (EAPC), with associated 95% confidence intervals derived from linear regression techniques (21). The analysis adhered to standard GBD 2021 methodologies, specifically employing DisMod-MR 2.1 modeling to integrate YLDs and DALYs while maintaining internal consistency across age-sex-location strata and utilizing Monte Carlo simulations with 1,000 iterations to propagate uncertainty from input data, model selection, and covariates throughout all estimation stages.

Statistical computations utilized R Studio 4.1.1 (R Project for Statistical Computing), while visual refinements were executed in Adobe Illustrator 2022.

## Results

### Global, regional, and national burden of childhood otitis media due to SHS exposure

The global YLDs due to childhood otitis media attributable to SHS exposure decreased by 17.65%, from 95,504 (95% UI: 42,399–169,078) years cases to 78,648 (95% UI: 34,414–142,066) years in 1990–2021. The ASYR declined by 28.78%, from 5.49 (95% UI: 2.38–9.9) per 100,000 population to 3.91 (95% UI: 1.66–7.23) per 100,000 population. Similarly, DALYs decreased by 21.59%, from 101,472 (95% UI: 46,038–178,276) to 79,560 (95% UI: 34,630–143,322) cases. The ASDR declined by 32.08%, from 5.83 (95% UI: 2.61–10.37) per 100,000 population to 3.96 (95% UI: 1.68–7.31) per 100,000 population. Overall, the burden of YLDs, DALYs, ASYR, and ASDR was higher in males than females. In 2021, the highest number of YLDs and DALYs were observed in low-middle SDI regions, with 25,936 (95% UI: 11,392–47,954) and 26,004 (95% UI: 11,405–47,988) cases, respectively, while the highest ASYR and ASDR were observed in high-middle SDI regions, with values of 4.83 (95% UI: 2.07–8.97) and 4.83 (95% UI: 2.08–8.98), respectively. All five SDI regions demonstrated a declining trend in both ASYR and ASDR. In 21 GBD regions, YLDs and DALYs decreased in all regions except Central Asia, Central Sub-Saharan Africa, Eastern Sub-Saharan Africa, North Africa and the Middle East, Oceania, and Western Sub-Saharan Africa. In contrast, ASYR and ASDR increased only in Oceania, with declines observed in all other regions. Notably, Central Sub-Saharan Africa exhibited the most substantial increase in YLDs and DALYs due to SHS exposure, rising to 1,458 (95% UI: 614–2,685) and 1,473 (95% UI: 623–2,695) years in 2021, respectively. Regional trends showed distinct patterns: Except for Oceania, all other regions demonstrated significant declines in both ASYR and ASDR (Appendix Table 1).

**Figure 1:**
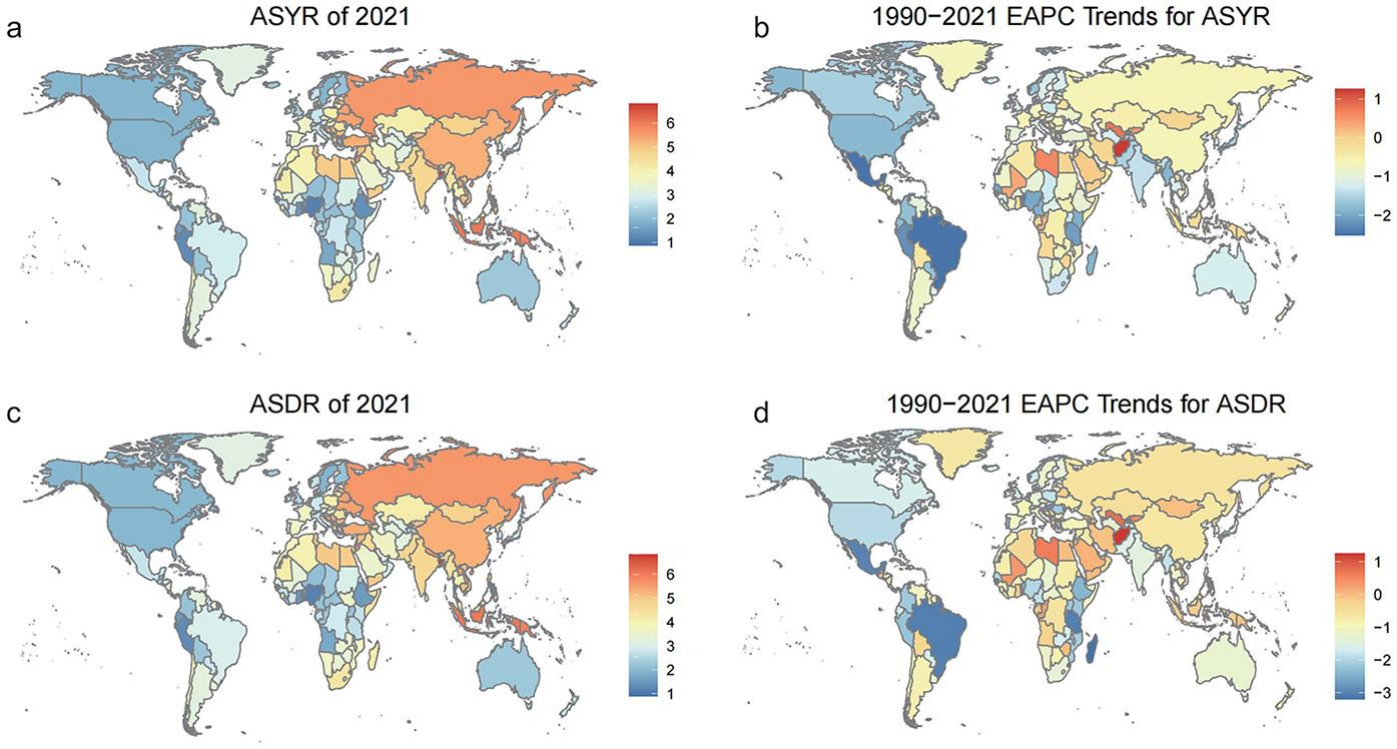
The childhood otitis media burden attributable to SHS across 204 countries and territories globally. **(a)** Age-standardized YLD rates (ASYR) in 2021, shown with a color gradient from blue (lower rates) to red (higher rates). **(b)** Estimated annual percentage change (EAPC) in ASYR from 1990 to 2021, with gradient from blue (decreasing trends) to red (increasing trends). **(c)** Age-standardized DALY rates (ASDR) in 2021, shown with a color gradient from blue (lower rates) to red (higher rates). **(d)** Estimated annual percentage change (EAPC) in ASDR from 1990 to 2021, with gradient from blue (decreasing trends) to red (increasing trends).

In 2021, the childhood otitis media burden attributable to SHS, as indicated by ASYR and ASDR, was highest in Southeast Asia (Bangladesh, Indonesia, Malaysia), Oceania (Papua New Guinea), Eastern Europe (Russia), and China. In contrast, countries in Africa (Ethiopia, Nigeria) and South America (Peru) reported lower ASYR and ASDR values. The EAPC for ASYR and ASDR due to childhood otitis media attributable to SHS showed a declining trend in most regions globally, with only a few regions exhibiting an increasing trend. Afghanistan recorded the highest increase, with an EAPC of 1.2534 for ASYR and 1.2529 for ASDR, respectively (Figure 1).

### Joinpoint regression analysis

**Figure 2:**
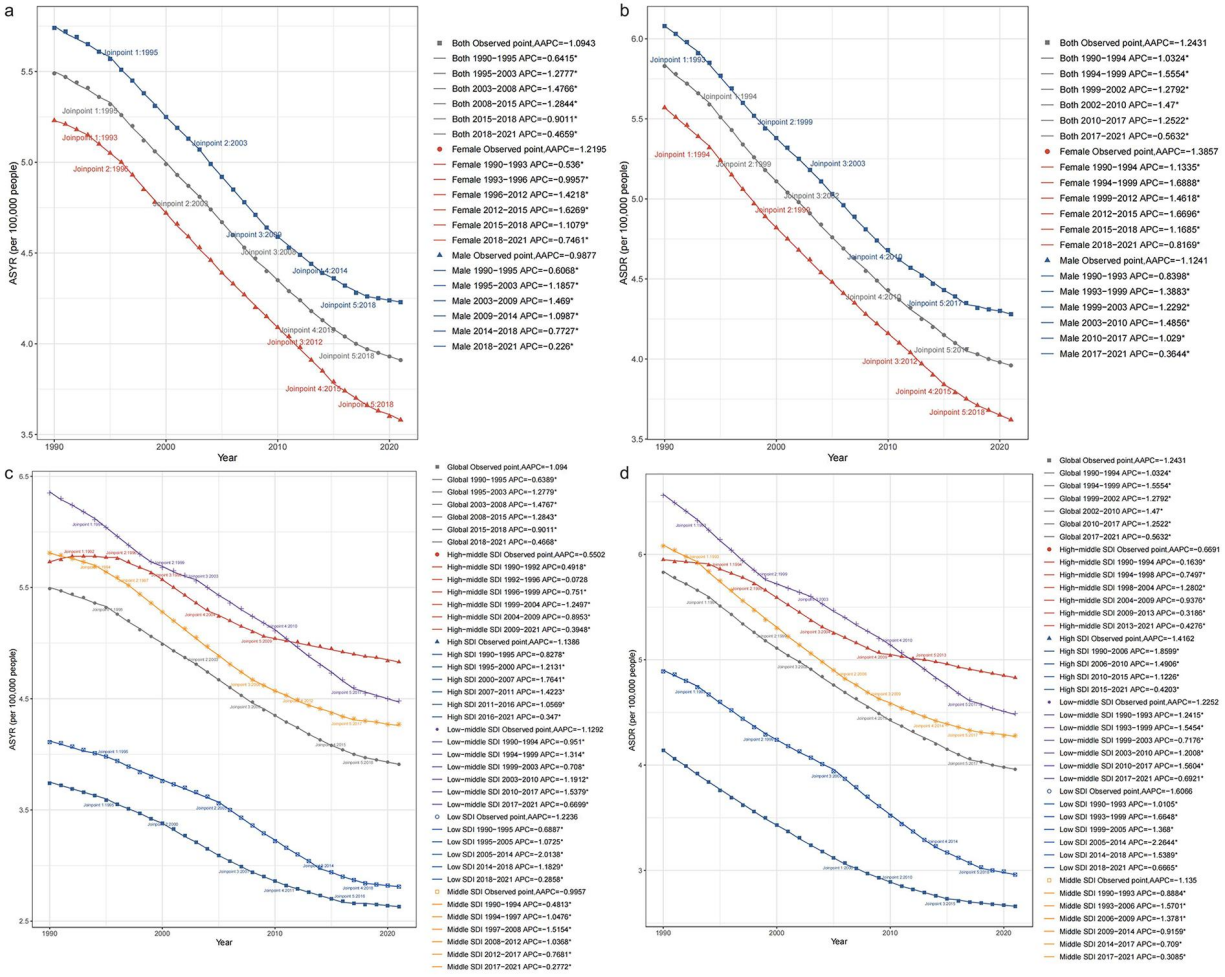
Trends in age-standardized rates (ASR) of ASYR and ASDR for childhood otitis media attributable to SHS, 1990–2021. **(a)** Comparative trends of ASYR and ASDR for both genders, with plotted data points and annotated annual percentage change (APC) values. **(b)** Separate trends of ASYR and ASDR for males and females, with labeled joinpoints indicating significant inflection years. **(c)** Global trends of ASYR and ASDR stratified by Socio-Demographic Index (SDI) levels (from high to low), showing variation across SDI categories. **(d)** Detailed ASYR and ASDR changes over time across global and SDI categories, with highlighted joinpoints showing major trend shifts.

The ASYR and ASDR for childhood otitis media attributable to SHS demonstrated a continuous decline globally in 1990–2021, as well as across the five SDI regions, for both gender. Notably, the most significant decline in global ASYR occurred in 2003–2008 [annual percentage change (APC): −1.48], while the most substantial decline in ASDR was observed between 1994 and 1999, with an APC of −1.56 (Figure 2).

### Age trends

**Figure 3:**
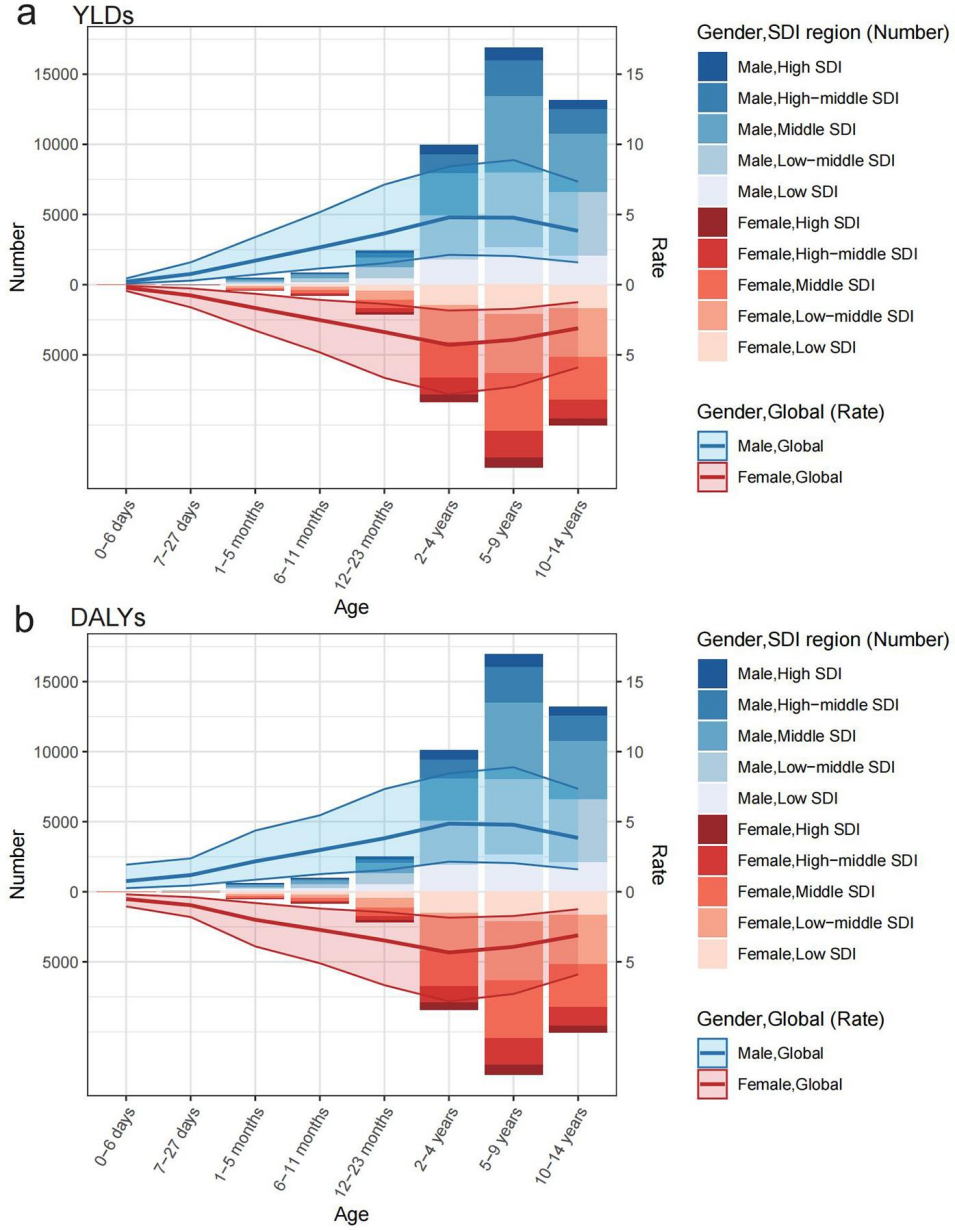
Age-specific burden of YLDs and DALYs attributable to SHS exposure in childhood otitis media, by gender and SDI region, 2021. **(a)** YLDs and **(b)** DALYs.

The YLDs and DALYs peaked between the ages of 5 and 9 years for both males and females, with males consistently exhibiting higher YLDs than females. The burden of YLDs and DALYs was higher in countries within the middle and low-middle SDI regions. Among males, the highest number of YLDs (5,416.62) and DALYs (5,422.12) occurred in the middle SDI regions at ages 5–9 years, while females experienced highest number of YLDs (4,216.23) and DALYs (4,218.74) in the low-middle SDI regions. The YLDs and DALYs rates increased initially and then decreased with age, with males reaching their peak YLDs and DALYs rates at ages 5–9 years, while females peaked at ages 2–4 years (Figure 3).

### Health inequality analysis

**Figure 4:**
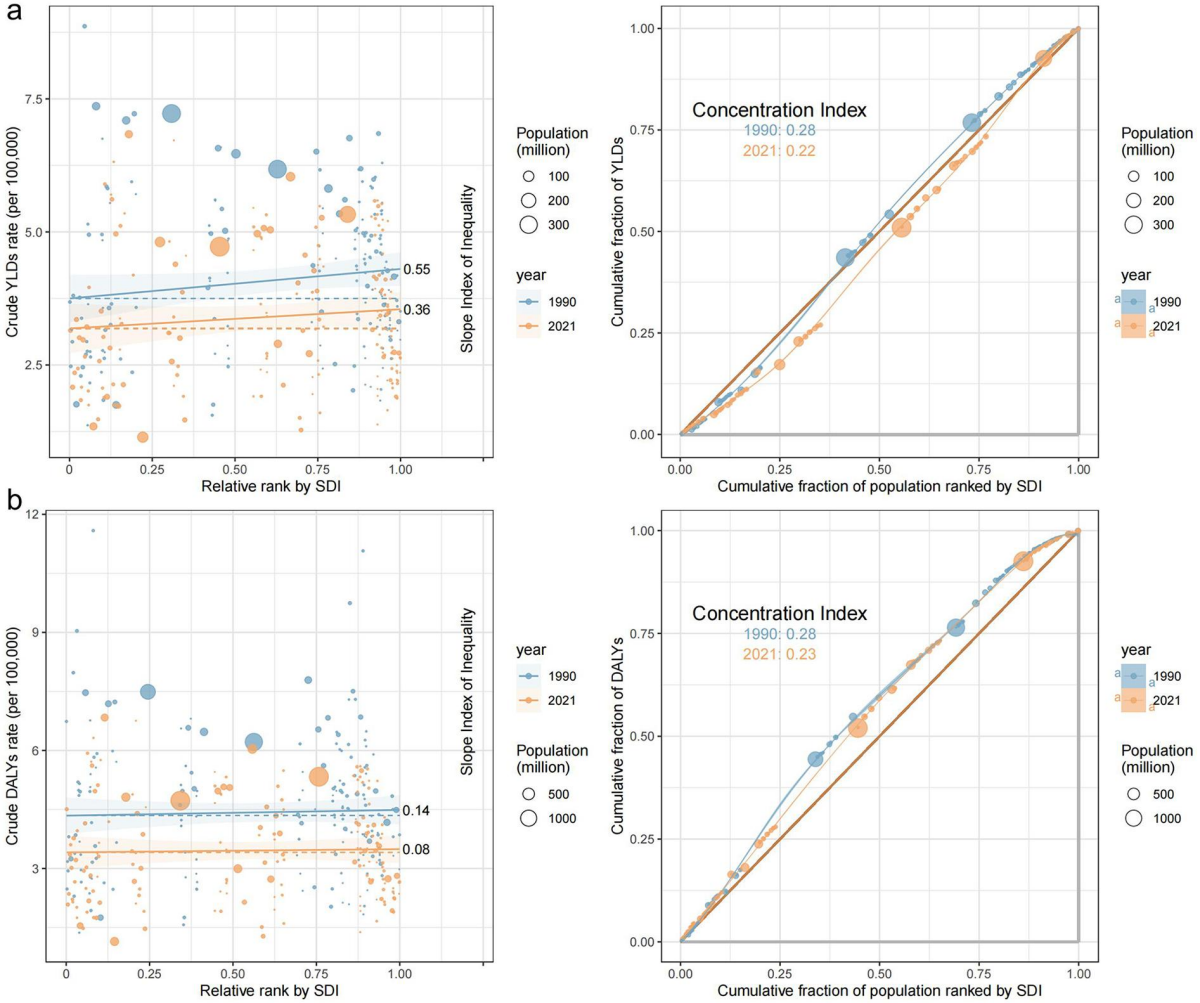
Health inequality metrics for childhood otitis media burden attributable to SHS, 1990 and 2021. **(a)** YLDs and **(b)** DALYs.

As the SDI increased, the YLDs and DALYs rates for childhood otitis media attributable to SHS showed an upward trend, likely reflecting higher diagnostic capacity and better reporting in high-SDI regions rather than a direct causal effect of SDI on disease burden. Both 1990 and 2021 exhibited some degree of inequality, although the curve in 1990 was slightly higher. The SII for YLDs and DALYs rates decreased from 0.55 and 0.14 in 1990 to 0.36 and 0.08 in 2021, respectively. Similarly, the CI for both YLDs and DALYs rates decreased from 0.28 in 1990 to 0.22 and 0.23 in 2021, respectively, suggesting a reduction in inequality in 2021 compared to 1990 (Figure 4).

### Future prediction

**Figure 5:**
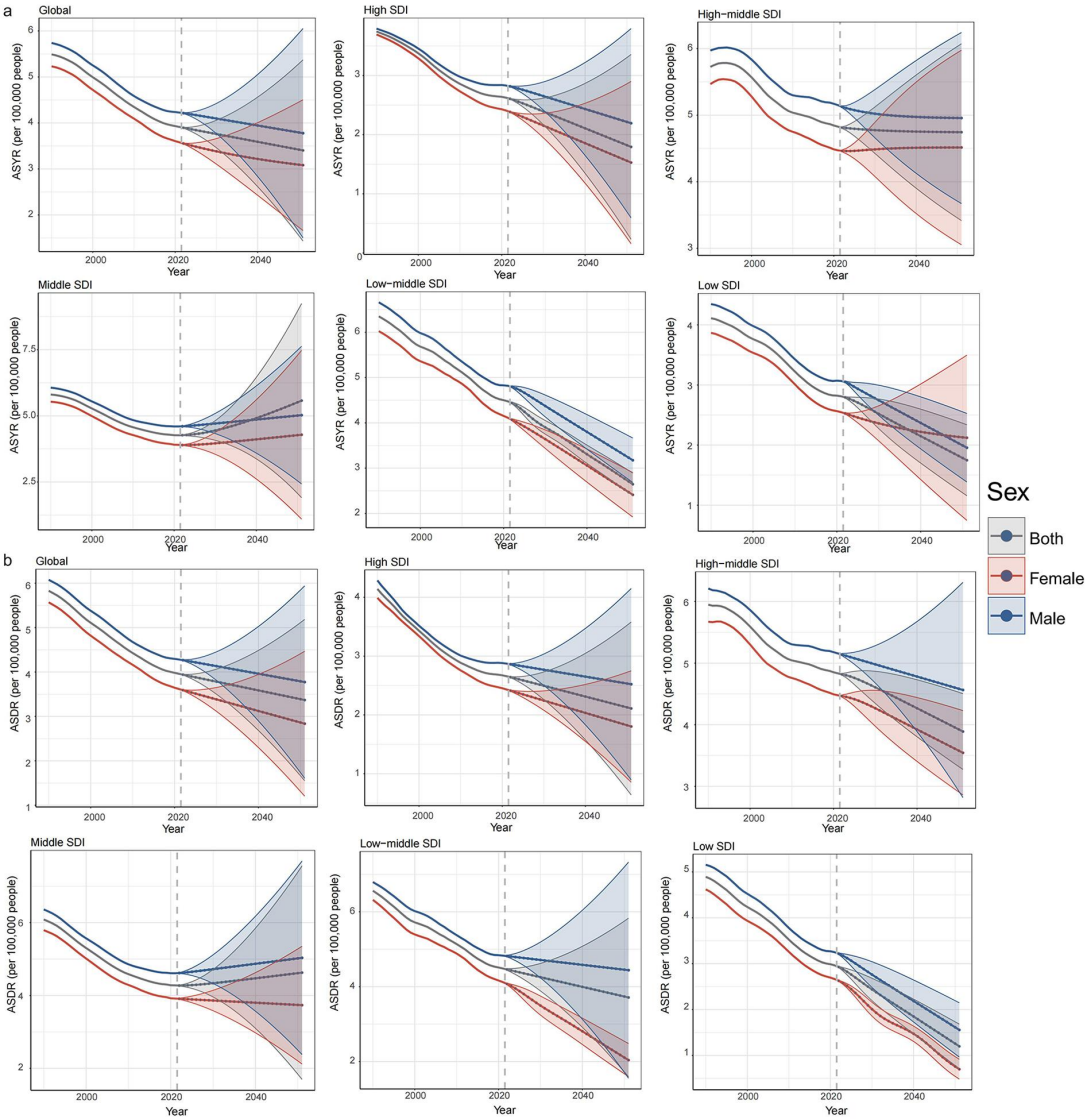
Projected age-standardized rates (ASR) of ASYR and ASDR for childhood otitis media attributable to SHS, 1990–2040. **(a)** ASYR and **(b)** ASDR.

The ASYR and ASDR for childhood otitis media attributable to SHS have exhibited a declining trend globally over the past 30 years, and this trend is expected to continue in the future (Figure 5). By 2051, the ASYR and ASDR are projected to decrease to 3.40 and 3.37 per 100,000, respectively. For males, these rates are expected to decrease to 3.78 and 3.77 per 100,000, while for females, they are projected to decline to 3.08 and 2.84 per 100,000. A similar trend is anticipated across high SDI, high-middle SDI, low-middle SDI, and low SDI regions. Among these four SDI regions, the most significant decline in ASYR for both sexes, males, and females is expected in the low-middle SDI regions, where rates are projected to decrease to 2.64, 3.17, and 2.41 per 100,000 by 2051, respectively, compared to 2021. The most pronounced decline in ASDR by 2051 is anticipated in low SDI regions for both sexes and for males specifically, with projected rates falling to 1.20 and 1.56 per 100,000, respectively. Female populations in low-middle SDI territories are anticipated to demonstrate the most substantial ASDR decline, reaching 0.70 cases per 100,000 individuals. Conversely, middle SDI regions are predicted to exhibit progressive increases in both ASYR and ASDR metrics across all demographic sex categories throughout the 30-year projection period. By 2051, the ASYR in these regions is estimated to reach 5.56 per 100,000 for both sexes, 5.03 per 100,000 for males, and 4.29 per 100,000 for females. Concurrently, the ASDR is projected to increase to 4.63 per 100,000 for both sexes and 5.03 per 100,000 for males. However, the ASDR for females in the middle SDI regions is projected to slightly decrease by 2051, reaching 3.73 per 100,000.

## Discussion

Previous GBD analyses have primarily concentrated on the global or regional burden of otitis media. However, studies focusing on the burden of otitis media attributable to specific risk factors, particularly in targeted populations, have been relatively limited. This study represents the first comprehensive epidemiological analysis of childhood otitis media using GBD 2021 data, examine the epidemiological characteristics of childhood otitis media attributable to SHS, including cross-regional, cross-country, and age-group comparisons.

From 1990 to 2021, the global burden of childhood otitis media attributable to SHS exposure has decreased. However, further analysis reveals that regions such as Central Asia, sub-Saharan Africa, North Africa, the Middle East, and Oceania have not experienced significant improvement in disease burden. These findings align with previous research showing persistent SHS exposure disparities in low-income populations (22), particularly in regions with weaker tobacco control policies. Notably, although the global burden has generally declined, low SDI regions have seen limited progress. Smoking remains a major global health issue, with cigarette smoke containing over 9,500 chemicals, many of which are harmful to human health. The U.S. Food and Drug Administration (FDA) identified 79 carcinogenic substances in tobacco smoke in its 2012 report (23). Beyond its established links to cancer and respiratory diseases, smoking also exerts a significant impact on the auditory system. A longitudinal cohort study by Hu et al. demonstrated dose-dependent increases in high- and low-frequency hearing loss risks with daily cigarette consumption, while highlighting that smoking-related excess hearing loss risks abate within a relatively short timeframe following cessation (24). For example, in the UK, approximately 130,200 cases of childhood otitis media occur annually, while in the U.S., 292,950 cases are attributed to household SHS exposure (25). A sustained global reduction in SHS-associated pediatric middle ear disease burden was observed from 1990 to 2021, with YLDs demonstrating 17.65% reduction and DALYs showing 21.59% decline. Corresponding age-standardized metrics exhibited more pronounced decreases: ASYR fell by 28.78% while ASDR dropped 32.08%, demonstrating correlation with intensified worldwide tobacco regulation initiatives. Notably, the introduction of the WHO Framework Convention on Tobacco Control (FCTC) facilitated decreased SHS exposure through policy interventions including smoke-free public space legislation, elevated tobacco taxation, and enhanced cessation support systems (26–28). The accelerated decline during 2003–2008 likely reflects combined effects of early FCTC adoption (with 2–3 year lag for measurable impacts, as shown in Tobacco Control 2024) and expanding pneumococcal vaccination programs (29). However, persistent epidemiological challenges continue in specific geographic zones, particularly Central Asia, sub-Saharan Africa, North African territories, Middle Eastern countries, and Oceanian regions. This is largely attributable to limited enforcement of tobacco control regulations, deeply rooted smoking norms, and sustained high levels of SHS exposure. In particular, countries such as Afghanistan, Kazakhstan, Kyrgyzstan, Libya, and Mali have experienced an upward trend in disease burden. These increases may reflect the combined effects of inadequate regulatory implementation, constrained healthcare infrastructure, lower socioeconomic development, and insufficient public awareness regarding SHS-related health risks. These factors further amplify the impact of SHS on child health in these regions.

Between 1990 and 2021, the burden of childhood otitis media attributable to SHS consistently declined across all gender groups. However, male children consistently experienced higher YLDs, DALYs, ASYR, and ASDR compared to females. The reasons for this gender difference are likely multifactorial, including possible biological susceptibility and differences in exposure patterns. Due to limitations of the GBD data. Future studies with more detailed exposure assessments, including time-activity patterns and biomarker measurements, are needed to clarify the drivers of this disparity (30, 31). Additionally, gender differences in disease burden could be influenced by healthcare-seeking behavior (32). Female children are typically brought to healthcare facilities earlier by their parents, which may mitigate the long-term impacts of the disease (33, 34). In contrast, male children may experience delayed medical attention, potentially leading to worsened disease outcomes. Estrogen and its associated signaling pathways might also play a role in protecting females from hearing loss (35, 36). Further analysis highlights that the 5–9 years group bears the highest burden of otitis media, such as the shorter, more horizontal orientation and poorer functional efficiency of the Eustachian tube—along with structural abnormalities including adenoid hypertrophy and craniofacial malformations (e.g., cleft palate or Down syndrome), all of which increase susceptibility to infections (32, 37). Studies have demonstrated that among children exposed to secondhand smoke, tobacco smoke exposure is significantly associated with recurrent acute otitis media (RAOM), otitis media with effusion (OME), and sensorineural hearing loss (SNHL) (38). Consequently, controlling SHS exposure, especially in this vulnerable age group, is essential for reducing the burden of childhood otitis media.

Our study also found that high SDI regions exhibited higher YLDs and DALYs rates due to SHS exposure. This pattern may partially reflect differential diagnostic capacity, as WHO HEN reports document 3–5 times greater otoscopy availability in high SDI regions compared to low-SDI settings, while studies estimate 40%–60% of otitis media cases go undiagnosed in resource-limited areas (39–41). Despite the implementation of stricter tobacco control policies in these regions, deeply ingrained tobacco consumption cultures and persistently high SHS exposure rates remain significant contributors to the burden (42). Additionally, the stronger medical diagnostic capabilities in high SDI regions lead to higher detection rates of childhood otitis media, which in turn increases the statistical burden of the disease (42). These diagnostic disparities suggest our estimates may underrepresent true global disparities in SHS exposure impacts. In contrast, low SDI regions currently exhibit a relatively lower disease burden, which may be attributed to lower tobacco usage rates, limited diagnostic capacity, and possible underreporting of cases (43). Although disparities in health outcomes have diminished from 1990 to 2021, these improvements likely stem from the global advancement of tobacco control initiatives. With ongoing economic development, medical infrastructure and service delivery in low SDI regions are gradually improving, facilitating more timely diagnosis and management of childhood otitis media, and potentially mitigating the disease's long-term consequences (44). Nevertheless, despite measurable progress in reducing inequality, additional efforts are still required to further reduce the burden of childhood otitis media linked to SHS exposure. Focused tobacco control interventions and equitable healthcare resource allocation remain pivotal to addressing persistent disparities across regions (45).

Global projections indicate a continued decline in the disease burden of childhood otitis media attributable to SHS over the next three decades. However, an upward trend in ASDR is anticipated in some middle SDI regions and individual countries, possibly due to rising tobacco consumption, weak policy implementation, and limited public awareness (45). Although global tobacco control strategies have advanced considerably, certain low- and middle-income regions need to reinforce regulatory frameworks, enforce smoke-free environments, and implement stricter controls on SHS exposure to achieve sustained progress (46). In this regard, strengthened international collaboration and financial assistance are crucial to supporting tobacco control and healthcare improvements in resource-constrained countries. Such concerted efforts are vital for meaningfully reducing the burden of childhood otitis media on a global scale (47). Future studies with more detailed exposure assessment are needed to better understand gender-specific risks.

This study has some limitations. First, due to the inherent time lag in the GBD data, the findings may not fully reflect the most recent changes in disease patterns. Additionally, the aggregation of acute and chronic otitis media within the GBD dataset prevents differentiation between these subtypes, which represents a notable constraint. Second, disparities in diagnostic capability across countries may lead to underdiagnosis, particularly in areas with inadequate healthcare infrastructure, potentially resulting in an underestimation of the true burden. Third, our exposure assessment, while adhering to the standardized GBD framework, possesses inherent limitations. The dichotomous definition of SHS exposure (exposed/unexposed) fails to capture critical variations in exposure source, intensity, duration, and timing. Moreover, the use of DALYs and YLDs as the primary outcome metric, while informative for overall burden estimation, precludes the calculation of attributable case numbers. Fourth, in regions experiencing political instability or humanitarian crises (e.g., Afghanistan), the reliability of health surveillance data may be compromised, requiring cautious interpretation of trends. Nonetheless, the comprehensive scope of GBD 2021 data—covering diverse countries, territories, and population groups—offers a valuable overview of the temporal and geographic trends in disease burden across different socioeconomic strata and between sexes. This broad perspective enhances the generalizability of the findings and supports evidence-informed policymaking at the global level. Moreover, by focusing on the burden of SHS-related childhood otitis media, this study fills an important gap in existing literature and provides a robust scientific foundation for developing more precise tobacco control strategies. The long-term projections included also offer critical direction for future interventions aimed at narrowing regional, gender-based, and age-specific disparities in disease outcomes.

## Conclusion

In 1990–2021, there was an overall downward trend in the global burden of childhood otitis media linked to SHS exposure. Nevertheless, some regions, such as Central Asia and Sub-Saharan Africa, did not exhibit substantial improvements. The disease consistently imposed a greater burden on male children, with those aged 5–9 years being the most affected group. Although international tobacco control initiatives have progressed, SHS exposure and tobacco use continue to pose considerable public health risks, particularly in low and some middle SDI settings. To achieve further reductions in the disease burden, it is crucial to reinforce tobacco control strategies, promote widespread public health education, and improve the equitable allocation of healthcare resources. Future research should also focus on strengthening international collaboration, optimizing surveillance systems, and developing training programmes for healthcare providers to enhance early detection and management. These measures are essential to protecting children's health and minimizing the long-term consequences of SHS exposure at a global level.

## Data Availability

The original contributions presented in the study are included in the article/Supplementary Material, further inquiries can be directed to the corresponding author.

## References

[1] Pichichero ME. Otitis media. Pediatr Clin N Am. (2013) 60(2):391–407. 10.1016/j.pcl.2012.12.007 23481107

[2] Alkan Ö Ünver Ş. Tobacco smoke exposure among women in Turkey and determinants. J Subst Use. (2022) 27(1):43–9. 10.1080/14659891.2021.1885518

[3] Ünver Ş Tekmanli HH Alkan Ö. Passive smoking as a risk factor among older adults: an ordered probability approach for Türkiye. Front Public Health. (2023) 11:1142635. 10.3389/fpubh.2023.1142635 37397727 PMC10310956

[4] Goulioumis AK Gkorpa M Athanasopoulos M Athanasopoulos I Gyftopoulos K. The Eustachian tube dysfunction in children: anatomical considerations and current trends in invasive therapeutic approaches. Cureus. (2022) 14(7):e27193. 10.7759/cureus.27193 36039214 PMC9395912

[5] Klimek L Brough HA Arasi S Toppila-Salmi S Bergmann C Jutel M Otitis media with effusion (OME) and Eustachian tube dysfunction: the role of allergy and immunity-an EAACI position paper. Allergy. (2025) 80(9):2429–41. 10.1111/all.16554 40242889

[6] GBD 2021 Upper Respiratory Infections Otitis Media Collaborators. Global, regional, and national burden of upper respiratory infections and otitis media, 1990–2021: a systematic analysis from the global burden of disease study 2021. Lancet Infect Dis. (2025) 25(1):36–51. 10.1016/S1473-3099(24)00430-4 39265593 PMC11680489

[7] Guo Z Ji W Song P Zhao J Yan M Zou X Global, regional, and national burden of hearing loss in children and adolescents, 1990–2021: a systematic analysis from the global burden of disease study 2021. BMC Public Health. (2024) 24(1):2521. 10.1186/s12889-024-20010-0 39285386 PMC11406738

[8] Danishyar A Ashurst JV. Acute Otitis Media [Internet]. Treasure Island (FL): StatPearls Publishing (2023). Available online at: http://www.ncbi.nlm.nih.gov/books/NBK470332/ (Accessed October 15, 2024). 29262176

[9] Heikkinen T Chonmaitree T. Importance of respiratory viruses in acute otitis media. Clin Microbiol Rev. (2003) 16(2):230–41. 10.1128/CMR.16.2.230-241.2003 12692096 PMC153141

[10] Leichtle A Lai Y Wollenberg B Wasserman SI Ryan AF. Innate signaling in otitis media: pathogenesis and recovery. Curr Allergy Asthma Rep. (2011) 11(1):78–84. 10.1007/s11882-010-0158-3 21049294 PMC3020300

[11] Flor LS Anderson JA Ahmad N Aravkin A Carr S Dai X Health effects associated with exposure to secondhand smoke: a burden of proof study. Nat Med. (2024) 30(1):149–67. 10.1038/s41591-023-02743-4 38195750 PMC10803272

[12] Lee HM Son YS Kim HS Kim JY Kim SH Lee JH Effects of particulate matter exposure on the Eustachian tube and middle ear Mucosa of rats. Clin Exp Otorhinolaryngol. (2023) 16(3):225–35. 10.21053/ceo.2023.00227 37202348 PMC10471908

[13] Leung AKC Wong AHC. Acute otitis media in children. Recent Pat Inflammation Allergy Drug Discovery. (2017) 11(1):32–40. 10.2174/1874609810666170712145332 28707578

[14] Wang CP Ma SJ Xu XF Wang JF Mei CZ Yang GH. The prevalence of household second-hand smoke exposure and its correlated factors in six counties of China. Tob Control. (2009) 18(2):121–6. 10.1136/tc.2008.024836 19131456 PMC2655043

[15] GBD 2021 Diseases and Injuries Collaborators. Global incidence, prevalence, years lived with disability (YLDs), disability-adjusted life-years (DALYs), and healthy life expectancy (HALE) for 371 diseases and injuries in 204 countries and territories and 811 subnational locations, 1990–2021: a systematic analysis for the global burden of disease study 2021. Lancet. (2024) 403(10440):2133–61. 10.1016/S0140-6736(24)00757-8 38642570 PMC11122111

[16] Xu Y Gong M Wang Y Yang Y Liu S Zeng Q. Global trends and forecasts of breast cancer incidence and deaths. Sci Data. (2023) 10(1):334. 10.1038/s41597-023-02253-5 37244901 PMC10224917

[17] GBD 2021 Stroke Risk Factor Collaborators. Global, regional, and national burden of stroke and its risk factors, 1990–2021: a systematic analysis for the global burden of disease study 2021. Lancet Neurol. (2024) 23(10):973–1003. 10.1016/S1474-4422(24)00369-7 39304265 PMC12254192

[18] Chen HS Zeichner S Anderson RN Espey DK Kim HJ Feuer EJ. The joinpoint-jump and joinpoint-comparability ratio model for trend analysis with applications to coding changes in health statistics. J Off Stat. (2020) 36(1):49–62. 10.2478/jos-2020-0003 32713989 PMC7380682

[19] Bai J Zhao Y Yang D Ma Y Yu C. Secular trends in chronic respiratory diseases mortality in Brazil, Russia, China, and South Africa: a comparative study across main BRICS countries from 1990 to 2019. BMC Public Health. (2022) 22(1):91. 10.1186/s12889-021-12484-z 35027030 PMC8759233

[20] ArunKumar KE Kalaga DV Sai Kumar CM Chilkoor G Kawaji M Brenza TM. Forecasting the dynamics of cumulative COVID-19 cases (confirmed, recovered and deaths) for top-16 countries using statistical machine learning models: auto-regressive integrated moving average (ARIMA) and seasonal auto-regressive integrated moving average (SARIMA). Appl Soft Comput. (2021) 103:107161. 10.1016/j.asoc.2021.107161 33584158 PMC7869631

[21] Yang X Chen H Zhang T Yin X Man J He Q Global, regional, and national burden of blindness and vision loss due to common eye diseases along with its attributable risk factors from 1990 to 2019: a systematic analysis from the global burden of disease study 2019. Aging. (2021) 13(15):19614–42. 10.18632/aging.203374 34371482 PMC8386528

[22] Alkan Ö Ünver Ş. Secondhand smoke exposure for different education levels: findings from a large, nationally representative survey in Turkey. BMJ Open. (2022) 12(2):e057360. 10.1136/bmjopen-2021-057360 35177464 PMC8860053

[23] Jones LL Hassanien A Cook DG Britton J Leonardi-Bee J. Parental smoking and the risk of middle ear disease in children: a systematic review and meta-analysis. Arch Pediatr Adolesc Med. (2012) 166(1):18–27. 10.1001/archpediatrics.2011.158 21893640

[24] Hu H Sasaki N Ogasawara T Nagahama S Akter S Kuwahara K Smoking, smoking cessation, and the risk of hearing loss: japan epidemiology collaboration on occupational health study. Nicotine Tob Res. (2019) 21(4):481–8. 10.1093/ntr/nty026 29547985

[25] GBD 2015 Tobacco Collaborators. Smoking prevalence and attributable disease burden in 195 countries and territories, 1990–2015: a systematic analysis from the global burden of disease study 2015. Lancet. (2017) 389(10082):1885–906. 10.1016/S0140-6736(17)30819-X 28390697 PMC5439023

[26] Bialous S Da Costa E Silva VL. Where next for the WHO framework convention on tobacco control? Tob Control. (2022) 31(2):183–6. 10.1136/tobaccocontrol-2021-056545 35241586

[27] Chan KH Xiao D Zhou M Peto R Chen Z. Tobacco control in China. Lancet Public Health. (2023) 8(12):e1006–15. 10.1016/S2468-2667(23)00242-6 38000880

[28] Cohen JE Myers ML Ahluwalia IB. WHO framework convention on tobacco control learnings. Health Secur. (2023) 21(5):428–9. 10.1089/hs.2023.0094 37552835 PMC10541915

[29] Paraje G Flores Muñoz M Wu DC Jha P. Reductions in smoking due to ratification of the framework convention for tobacco control in 171 countries. Nat Med. (2024) 30(3):683–9. 10.1038/s41591-024-02806-0 38321222 PMC10957467

[30] Jin Y Dong L Jiang Y Dong W Li Z Lu W Global burden and prevalence of otitis media-induced hearing loss in children: 32-year study. Eur Arch Otorhinolaryngol. (2025) 282(10):5189–99. 10.1007/s00405-025-09461-2 40419775 PMC12518489

[31] Su Z Xie Y Huang Z Cheng A Zhou X Wang M Second hand smoke attributable disease burden in 204 countries and territories, 1990–2021: a systematic analysis from the global burden of disease study 2021. Respir Res. (2025) 26(1):174. 10.1186/s12931-025-03228-3 40336093 PMC12060545

[32] Simon AK Hollander GA McMichael A. Evolution of the immune system in humans from infancy to old age. Proc Biol Sci. (2015) 282(1821):20143085. 10.1098/rspb.2014.3085 26702035 PMC4707740

[33] Bertakis KD Azari R Helms LJ Callahan EJ Robbins JA. Gender differences in the utilization of health care services. J Fam Pract. (2000) 49(2):147–52. 10718692

[34] Marom T Tan A Wilkinson GS Pierson KS Freeman JL Chonmaitree T. Trends in otitis media-related health care use in the United States, 2001–2011. JAMA Pediatr. (2014) 168(1):68–75. 10.1001/jamapediatrics.2013.3924 24276262 PMC3947317

[35] Shuster B Casserly R Lipford E Olszewski R Milon B Viechweg S Estradiol protects against noise-induced hearing loss and modulates auditory physiology in female mice. Int J Mol Sci. (2021) 22(22):12208. 10.3390/ijms222212208 34830090 PMC8620009

[36] Li XT Qiu XY. 17β-estradiol upregulated expression of α and β subunits of larger-conductance calcium-activated K(+) channels (BK) via estrogen receptor β. J Mol Neurosci. (2015) 56(4):799–807. 10.1007/s12031-015-0502-0 25676031

[37] Macintyre EA Karr CJ Koehoorn M Demers P Tamburic L Lencar C Otitis media incidence and risk factors in a population-based birth cohort. Paediatr Child Health. (2010) 15(7):437–42. 10.1093/pch/15.7.437 21886448 PMC2948776

[38] Patel S Wooles N Martin T. A systematic review of the impact of cigarettes and electronic cigarettes in otology. J Laryngol Otol. (2020) 4:1–6. 10.1017/S0022215120002315 33272335

[39] Heward E Saeed H Bate S Rajai A Molloy J Isba R Risk factors associated with the development of chronic suppurative otitis media in children: systematic review and meta-analysis. Clin Otolaryngol. (2024) 49(1):62–73. 10.1111/coa.14102 37794685

[40] Shen Y Zhou T Zou W Zhang J Yan S Ye H Global, regional, and national burden of hearing loss from 1990 to 2021: findings from the 2021 global burden of disease study. Ann Med. (2025) 57(1):2527367. 10.1080/07853890.2025.2527367 40605509 PMC12231259

[41] Kamenov K Martinez R Kunjumen T Chadha S. Ear and hearing care workforce: current Status and its implications. Ear Hear. (2021) 42(2):249–57. 10.1097/AUD.0000000000001007 33480624

[42] GBD 2016 Healthcare Access and Quality Collaborators. Measuring performance on the healthcare access and quality Index for 195 countries and territories and selected subnational locations: a systematic analysis from the global burden of disease study 2016. Lancet. (2018) 391(10136):2236–71. 10.1016/S0140-6736(18)30994-2 29893224 PMC5986687

[43] Monasta L Ronfani L Marchetti F Montico M Vecchi Brumatti L Bavcar A Burden of disease caused by otitis media: systematic review and global estimates. PLoS One. (2012) 7(4):e36226. 10.1371/journal.pone.0036226 22558393 PMC3340347

[44] World Health Organization. WHO Global Report on Trends in Prevalence of Tobacco Use 2000–2025. Geneva: World Health Organization (2021).

[45] Tauras JA Huang J Chaloupka FJ. Differential impact of tobacco control policies on youth sub-populations. Int J Environ Res Public Health. (2013) 10(9):4306–22. 10.3390/ijerph10094306 24036487 PMC3799499

[46] Tauras JA Chaloupka FJ Quah AC Fong GT. The economics of tobacco control: evidence from the international tobacco control (ITC) policy evaluation project. Tob Control. (2014) 23 Suppl 1(0 1):i1–3. 10.1136/tobaccocontrol-2014-051547 24500268 PMC4601579

[47] Jamison DT Alwan A Mock CN Nugent R Watkins D Adeyi O Universal health coverage and intersectoral action for health: key messages from disease control priorities, 3rd edition. Lancet. (2018) 391(10125):1108–20. 10.1016/S0140-6736(17)32906-9 29179954 PMC5996988

