# Supplementary material for: Global burden of childhood otitis media attributable to secondhand smoke from 1990 to 2021: a systematic analysis of the global burden of disease study 2021
Source: Front Pediatr. 2025 Dec 1;13:1619721. doi: 10.3389/fped.2025.1619721 (PMC12702889; doi:10.3389/fped.2025.1619721)
Supplement: Supplementary file 1 [file Supplementaryfile1.pdf]

# Secondhand smoke

## Flowchart

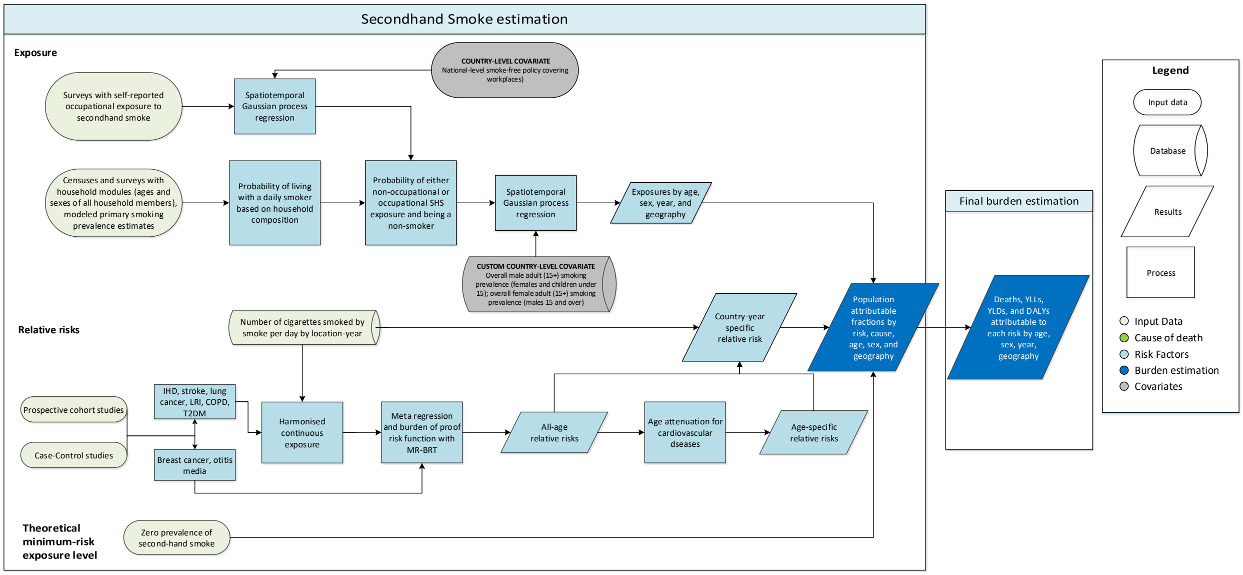


## Exposure

### Case definition

We define secondhand smoke exposure as current exposure to secondhand tobacco smoke at home or at work. We use household composition as a proxy for household secondhand smoke exposure and make the assumption that all persons living with a daily smoker are exposed to tobacco smoke. We use surveys to estimate the proportion of the population exposed to secondhand smoke at work. We only consider non-smokers to be exposed to secondhand smoke. Non-smokers are defined as all persons who are not daily smokers. Ex-smokers and occasional smokers are considered non-smokers in this analysis. Exposure is evaluated for both children and adults.

### Input data

To calculate the proportion of non-smokers who live with at least one daily smoker, two types of data were used: 1) unit record data on household composition, which included the ages and sexes of all persons living in the same household, and 2) GBD daily smoking estimates for each location, year, sex, and age group. Major survey series with a household composition module – including the Demographic Health Surveys (DHS), the Multiple Indicator Cluster Surveys (MICS), and the Living Standards Measurement Surveys (LSMS) – and national and subnational censuses, which included those captured in the Integrated Public Use Microdata Series (IPUMS) project, were used.

To calculate the proportion of the population exposed to secondhand smoke at work, by age and sex, we used cross-sectional surveys that ask respondents about self-reported occupational secondhand smoke exposure. Sources include the Global Adult Tobacco Surveys (GATS), Eurobarometer Surveys,

WHO Stepwise Approach to NCD Risk Factor Surveillance (STEPS) Surveys, and other regional and national survey series.

We updated our systematic review in GBD 2021 by searching the Global Health Data Exchange (GHDx) using the keywords “environmental tobacco smoke”, for workplace exposure, and “family composition”, for identifying household composition modules. We prioritised extraction of surveys used for estimating exposure at the workplace and of new household modules for filing in location and time gaps. Sources that reported exposure to secondhand smoke in a setting other than the workplace were not used. Due to the type of analysis performed, we restricted our data sources to those with available microdata (tabulated data-only sources were excluded). Given the nature of the data used in our models (microdata), no crosswalk for case definition adjustment or age and sex splitting processes were required. Table 1 provides a summary of the exposure input data.

**Table 1: Data inputs for exposure for secondhand smoke**

| | Countries with data | New sources | Total sources |
|---|---|---|---|
| Exposure | 176 | 480 | 1198 |

## Modelling strategy

Identical to GBD 2019, we estimated the probability that each person is living with a smoker and is also a non-smoker themselves using set theory. Household composition data were used at the individual level to capture the ages and sexes of each person in the household. In the past, we analyzed surveys with both household composition data and tobacco use questions and determined that the distribution of household size, mean age of the household members, and the age distribution were not significantly different between households with and without a self-reported smoker. Since we did not find that household composition varied between smokers and non-smokers, we then used the updated GBD 2021 daily smoking prevalence estimates to calculate the probability that each household member is a daily smoker. Next, we used the probability of the union of sets on each individual household member to calculate the overall probability that at least one of the other household members was a daily smoker.

As in GBD 2019, we incorporated occupational exposure by modelling prevalence of current exposure to secondhand smoke at work, by age, sex, location, and year, in a three-step spatiotemporal Gaussian process regression (ST-GPR), which generates exposure estimates from a mixed-effects hierarchical linear model plus weighted residuals smoothed across time, space, and age. For this, we first processed all data to capture exposure to secondhand smoke at work among anyone working primarily indoors. Using information from survey-specific gateway questions, we considered all those not currently working or not currently working primarily indoors not exposed to secondhand smoke.

The processed microdata was used to generate a complete time series from 1990 to 2022 for the proportion of the population exposed to tobacco smoke at the workplace using the ST-GPR. The first step of the ST-GPR process is a linear mixed-effects regression of our data on a set of potentially predictive covariates. In addition to the daily smoking prevalence estimates taken from the GBD study covariates database, in GBD 2021 we incorporated a dummy covariate to reflect if a national-level smoking ban covering workplaces was in place in each location-year. The data used to create this covariate came mainly from several iterations of the WHO report on the global tobacco epidemic.

With the estimated workplace exposure from ST-GPR, in order to avoid double counting, we calculated the probability that an individual is exposed through either household exposure or occupational exposure, given their age, sex, and household composition. Lastly, we multiplied this probability of exposure by the probability that the individual is not a smoker themselves (ie, 1 minus primary daily smoking prevalence for that person's location, year, age, and sex). We then collapsed these individual-level probabilities to produce average probabilities of exposure by location, year, age, and sex.

These final probabilities were modelled in the GBD ST-GPR framework. The linear model formula was fit separately by sex using restricted maximum likelihood in R. We used the sex-specific overall daily smoking prevalence for adults (age 15 and older) as a country-level covariate in the model. The overall male adult daily smoking prevalence was used as the covariate for females of all ages and for males under age 15. The overall female adult daily smoking prevalence was used as the covariate for males age 15 and older.

All input datapoints from the probability calculation had a measure of uncertainty (variance and sample size) coming from the uncertainty of the primary smoking prevalence model and the sample size from the unit record data going into the modelling process. Geographical random effects were used in model fitting but were not used in prediction.

## Theoretical minimum risk exposure level

The theoretical minimum risk exposure level for secondhand smoke is zero exposure among non-smokers, meaning that non-smokers would not live with any daily smokers and would not be exposed to tobacco smoke at their workplace.

## Relative risks

The same risk-outcome pairs from GBD 2019 were used. For children ages 0–14, we estimated the burden of otitis media attributable to secondhand smoke exposure. For all ages, we estimated the burden of lower respiratory infections (LRI) and for adults greater than or equal to 25 years of age, we estimated the burden of lung cancer, chronic obstructive pulmonary disease (COPD), ischaemic heart disease (IHD), ischaemic stroke, breast cancer, and type 2 diabetes (T2DM).

### Input data

In GBD 2021, we moved from deriving our relative risks from the integrated exposure response curves (IER) for $PM_{2.5}$ air pollution to creating relative risk curves using secondhand smoke-specific studies. We conducted an updated systematic review for studies published before December 31, 2019, evaluating the relationship between exposure to secondhand smoke and risk of IHD, stroke, COPD, breast cancer, and otitis media. We searched for studies in PubMed using the search strings reported in Table 2. Meta-analysis identified through our search were reviewed and underlying studies were considered for inclusion if not previously captured by our search strings. For the remaining outcomes – lung cancer, LRI, and T2DM –, we selected the secondhand smoke studies from the database that was used in GBD 2019 for generating the IER curve.

**Table 2: Search strings used to search PubMed database**

| Outcome | String |
|---|---|
| Ischaemic heart disease | (Tobacco smoke pollution [MeSH Terms] OR second-hand[Title/Abstract] OR secondhand[Title/Abstract] OR environmental tobacco[Title/Abstract] OR tobacco smoke[Title/Abstract] OR cigarette smoke[Title/Abstract] OR passive smok*[Title/Abstract] OR involuntary smok*[Title/Abstract] OR parental smoking[Title/Abstract] OR maternal smoking[Title/Abstract]) AND (Coronary Artery Disease[MeSH] OR Myocardial Ischemia[MeSH] OR atherosclerosis[MeSH] OR Coronary Artery Disease[Title/Abstract] OR Myocardial Ischemia[Title/Abstract] OR cardiac ischemia[Title/Abstract] OR silent ischemia[Title/Abstract] OR atherosclerosis [Title/Abstract] OR Ischaemic heart disease[Title/Abstract] OR Ischemic heart disease[Title/Abstract] OR coronary heart disease[Title/Abstract] OR myocardial infarction[Title/Abstract] OR heart attack[Title/Abstract] OR heart infarction[Title/Abstract]) AND (Case-Control Studies[MeSH Terms] OR Cross-Over Studies[MeSH Terms] OR Cohort Studies[MeSH Terms] OR Systematic Review[Publication Type] OR Meta-Analysis[Publication Type] OR “systematic review”[Title/Abstract] OR “meta-analysis”[Title/Abstract] OR “cohort”[Title/Abstract] OR “cross-over”[Title/Abstract] OR “crossover”[Title/Abstract] OR “case-control”[Title/Abstract] OR “prospective”[Title/Abstract] OR “retrospective”[Title/Abstract] OR “longitudinal”[Title/Abstract] OR “follow-up”[Title/Abstract] OR Dose-Response Relationship, Drug[MeSH Terms] OR “dose-response”[Title/Abstract]) AND (Risk[MeSH Terms] OR Odds Ratio[MeSH Terms] OR “risk”[Title/Abstract] OR “odds ratio”[Title/Abstract] OR “cross-product ratio”[Title/Abstract] OR “hazards ratio”[Title/Abstract] OR “hazard ratio”[Title/Abstract]) AND ("1970/01/01"[PDat] : "2019/12/31"[PDat]) AND (English[LA]) NOT (animals[MeSH Terms] NOT Humans[MeSH Terms]) |
| Ischaemic stroke | (Tobacco smoke pollution [MeSH Terms] OR second-hand[Title/Abstract] OR secondhand[Title/Abstract] OR environmental tobacco[Title/Abstract] OR tobacco smoke[Title/Abstract] OR cigarette smoke[Title/Abstract] OR passive smok*[Title/Abstract] OR involuntary smok*[Title/Abstract] OR parental smoking[Title/Abstract] OR maternal smoking[Title/Abstract]) AND (brain infarction[MeSH Terms] OR stroke[MeSH Terms] OR intracranial hemorrhages[MeSH Terms] OR "stroke"[Title/Abstract] OR “brain infarction”[Title/Abstract] OR “cerebral infarction”[Title/Abstract] OR “intracerebral hemorrhage”[Title/Abstract] OR “intracerebral haemorrhage”[Title/Abstract] OR “subarachnoid hemorrhage”[Title/Abstract] OR “subarachnoid haemorrhage”[Title/Abstract]) AND (Case-Control Studies[MeSH Terms] OR Cross-Over Studies[MeSH Terms] |

| | |
|---|---|
| | OR Cohort Studies[MeSH Terms] OR Systematic Review[Publication Type] OR Meta-Analysis[Publication Type] OR “systematic review”[Title/Abstract] OR “meta-analysis”[Title/Abstract] OR “cohort”[Title/Abstract] OR “cross-over”[Title/Abstract] OR “crossover”[Title/Abstract] OR “case-control”[Title/Abstract] OR “prospective”[Title/Abstract] OR “retrospective”[Title/Abstract] OR “longitudinal”[Title/Abstract] OR “follow-up”[Title/Abstract] OR Dose-Response Relationship, Drug[MeSH Terms] OR “dose-response”[Title/Abstract]) AND (Risk[MeSH Terms] OR Odds Ratio[MeSH Terms] OR “risk”[Title/Abstract] OR “odds ratio”[Title/Abstract] OR “cross-product ratio”[Title/Abstract] OR “hazards ratio”[Title/Abstract] OR “hazard ratio”[Title/Abstract]) AND ("1970/01/01"[PDat] : "2019/12/31"[PDat]) AND (English[LA]) NOT (animals[MeSH Terms] NOT Humans[MeSH Terms]) |
| Chronic obstructive pulmonary disease | (Tobacco smoke pollution [MeSH Terms] OR second-hand[Title/Abstract] OR secondhand[Title/Abstract] OR environmental tobacco[Title/Abstract] OR tobacco smoke[Title/Abstract] OR cigarette smoke[Title/Abstract] OR passive smok*[Title/Abstract] OR involuntary smok*[Title/Abstract] OR parental smoking[Title/Abstract] OR maternal smoking[Title/Abstract]) AND (Pulmonary Disease, Chronic Obstructive[MeSH] OR "COPD"[ Title/Abstract] OR "emphysema"[ Title/Abstract] OR "chronic obstructive pulmonary disease"[ Title/Abstract]) AND (Case-Control Studies[MeSH Terms] OR Cross-Over Studies[MeSH Terms] OR Cohort Studies[MeSH Terms] OR Systematic Review[Publication Type] OR Meta-Analysis[Publication Type] OR “systematic review”[Title/Abstract] OR “meta-analysis”[Title/Abstract] OR “cohort”[Title/Abstract] OR “cross-over”[Title/Abstract] OR “crossover”[Title/Abstract] OR “case-control”[Title/Abstract] OR “prospective”[Title/Abstract] OR “retrospective”[Title/Abstract] OR “longitudinal”[Title/Abstract] OR “follow-up”[Title/Abstract] OR Dose-Response Relationship, Drug[MeSH Terms] OR “dose-response”[Title/Abstract]) AND (Risk[MeSH Terms] OR Odds Ratio[MeSH Terms] OR “risk”[Title/Abstract] OR “odds ratio”[Title/Abstract] OR “cross-product ratio”[Title/Abstract] OR “hazards ratio”[Title/Abstract] OR “hazard ratio”[Title/Abstract]) AND ("1970/01/01"[PDat] : "2019/12/31"[PDat]) AND (English[LA]) NOT (animals[MeSH Terms] NOT Humans[MeSH Terms]) |

| | |
|---|---|
| Breast cancer | (Tobacco smoke pollution [MeSH Terms] OR second-hand[Title/Abstract] OR secondhand[Title/Abstract] OR environmental tobacco[Title/Abstract] OR tobacco smoke[Title/Abstract] OR cigarette smoke[Title/Abstract] OR passive smok*[Title/Abstract] OR involuntary smok*[Title/Abstract] OR parental smoking[Title/Abstract] OR maternal smoking[Title/Abstract]) AND (breast neoplasm[MeSH Terms] OR "breast cancer"[Title/Abstract] OR "breast cancers"[Title/Abstract] OR "breast neoplasm"[Title/Abstract] OR "breast neoplasms"[Title/Abstract] OR "mammary cancer"[MeSH Terms] OR "mammary cancers"[Title/Abstract] OR "breast malignant neoplasm"[Title/Abstract] OR "breast malignant neoplasms"[Title/Abstract] OR "mammary carcinoma"[Title/Abstract] OR "mammary carcinomas"[Title/Abstract] OR "breast carcinoma"[Title/Abstract] OR "breast carcinomas"[Title/Abstract] OR "mammary neoplasm"[Title/Abstract] OR "mammary neoplasms"[Title/Abstract] OR "breast tumor"[Title/Abstract] OR "breast tumors"[Title/Abstract] OR "cancer of the breast"[Title/Abstract] OR "cancers of the breast"[Title/Abstract] OR "neoplasm of the breast"[Title/Abstract] OR "tumor of the breast"[Title/Abstract]) AND (Case-Control Studies[MeSH Terms] OR Cross-Over Studies[MeSH Terms] OR Cohort Studies[MeSH Terms] OR Systematic Review[Publication Type] OR Meta-Analysis[Publication Type] OR "systematic review"[Title/Abstract] OR "meta-analysis"[Title/Abstract] OR "cohort"[Title/Abstract] OR "cross-over"[Title/Abstract] OR "crossover"[Title/Abstract] OR "case-control"[Title/Abstract] OR "prospective"[Title/Abstract] OR "retrospective"[Title/Abstract] OR "longitudinal"[Title/Abstract] OR "follow-up"[Title/Abstract] OR Dose-Response Relationship, Drug[MeSH Terms] OR "dose-response"[Title/Abstract]) AND (Risk[MeSH Terms] OR Odds Ratio[MeSH Terms] OR "risk"[Title/Abstract] OR "odds ratio"[Title/Abstract] OR "cross-product ratio"[Title/Abstract] OR "hazards ratio"[Title/Abstract] OR "hazard ratio"[Title/Abstract]) AND ("1970/01/01"[PDat] : "2019/12/31"[PDat]) AND (English[LA]) NOT (animals[MeSH Terms] NOT Humans[MeSH Terms]) |
| Otitis media | (Tobacco smoke pollution [MeSH Terms] OR second-hand[Title/Abstract] OR secondhand[Title/Abstract] OR environmental tobacco[Title/Abstract] OR tobacco smoke[Title/Abstract] OR cigarette smoke[Title/Abstract] OR passive smok*[Title/Abstract] OR involuntary smok*[Title/Abstract] OR parental smoking[Title/Abstract] OR maternal smoking[Title/Abstract]) AND (Otitis Media[MeSH Terms] OR "otitis media"[Title/Abstract] OR "middle ear infection" [Title/Abstract] OR "middle ear disease" [Title/Abstract] OR "ear infection"[Title/Abstract] OR "ear disease"[Title/Abstract] OR "otitis" [Title/Abstract]) AND (Case-Control Studies[MeSH Terms] OR Cross-Over Studies[MeSH Terms] OR Cohort Studies[MeSH Terms] OR Systematic Review[Publication Type] OR Meta-Analysis[Publication Type] OR "systematic review"[Title/Abstract] OR "meta-analysis"[Title/Abstract] |

| | |
|---|---|
| | OR “cohort”[Title/Abstract] OR “cross-over”[Title/Abstract] OR “crossover”[Title/Abstract] OR “case-control”[Title/Abstract] OR “prospective”[Title/Abstract] OR “retrospective”[Title/Abstract] OR “longitudinal”[Title/Abstract] OR “follow-up”[Title/Abstract] OR Dose-Response Relationship, Drug[MeSH Terms] OR “dose-response”[Title/Abstract]) AND (Risk[MeSH Terms] OR Odds Ratio[MeSH Terms] OR “risk”[Title/Abstract] OR “odds ratio”[Title/Abstract] OR “cross-product ratio”[Title/Abstract] OR “hazards ratio”[Title/Abstract] OR “hazard ratio”[Title/Abstract]) AND ("1970/01/01"[PDat] : "2019/12/31"[PDat]) AND (English[LA]) NOT (animals[MeSH Terms] NOT Humans[MeSH Terms]) |

PRISMA 2020 flow diagram for a new systematic review of the secondhand smoke and ischemic heart disease risk-outcome pair in GBD 2021

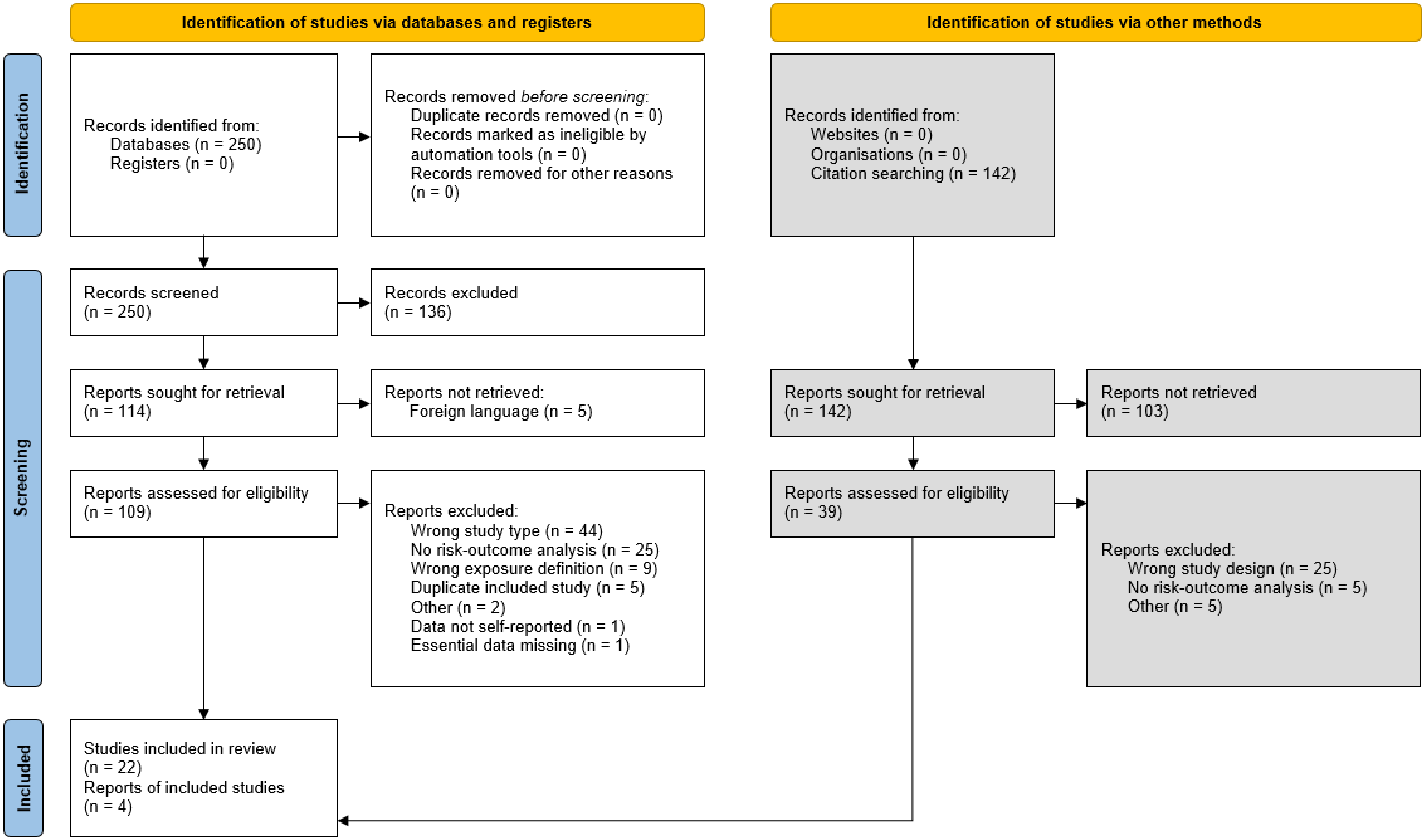


*From:* Page MJ, McKenzie JE, Bossuyt PM, Boutron I, Hoffmann TC, Mulrow CD, et al. The PRISMA 2020 statement: an updated guideline for reporting systematic reviews. BMJ 2021;372:n71. doi: 10.1136/bmj.n71. For more information, visit: http://www.prisma-statement.org/

**PRISMA 2020 flow diagram for a new systematic review of the secondhand smoke and ischaemic stroke risk-outcome pair in GBD 2021**

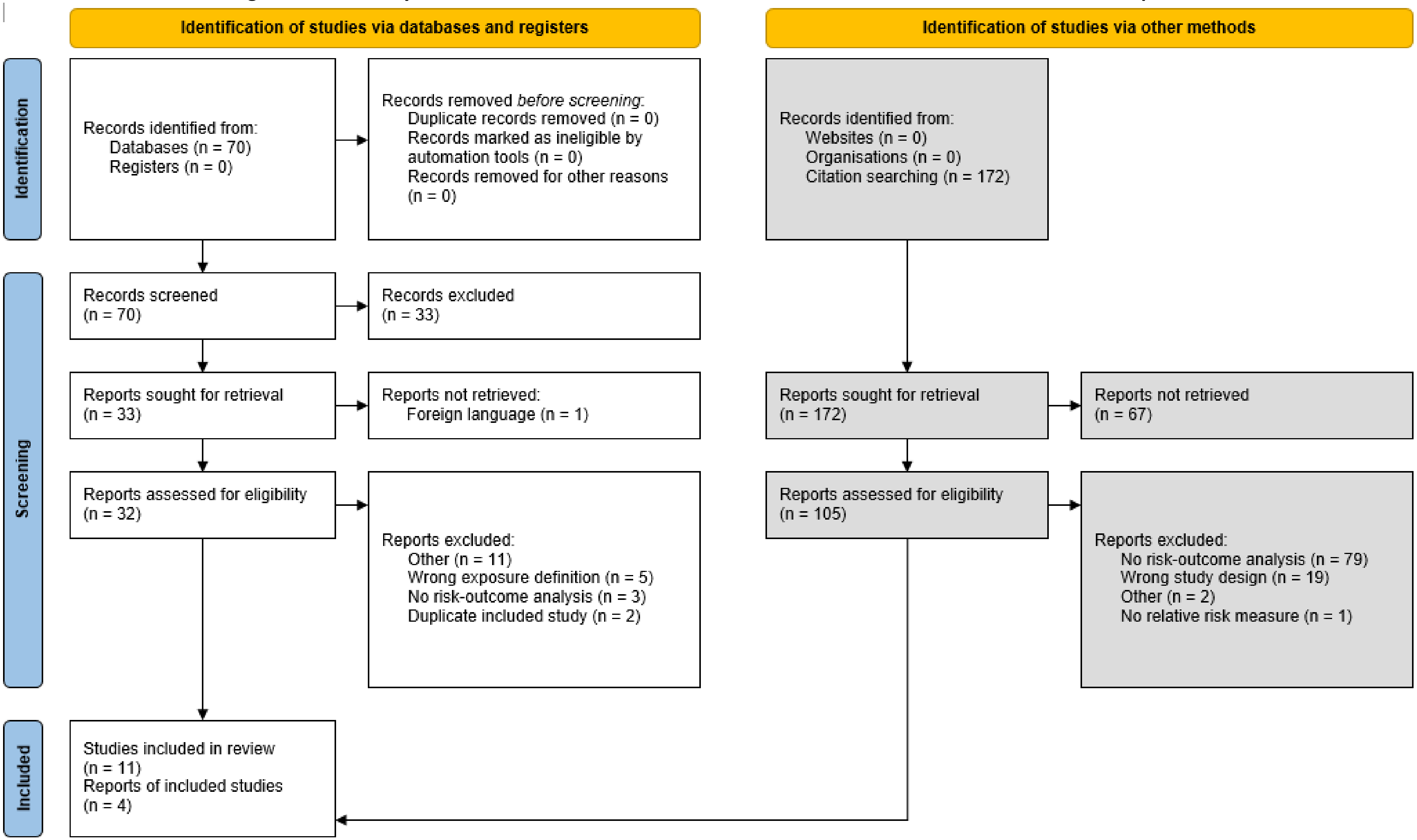


*From:* Page MJ, McKenzie JE, Bossuyt PM, Boutron I, Hoffmann TC, Mulrow CD, et al. The PRISMA 2020 statement: an updated guideline for reporting systematic reviews. BMJ 2021;372:n71. doi: 10.1136/bmj.n71. For more information, visit: http://www.prisma-statement.org/

**PRISMA 2020 flow diagram for a new systematic review of the secondhand smoke and chronic obstructive pulmonary disease risk-outcome pair in GBD 2021**

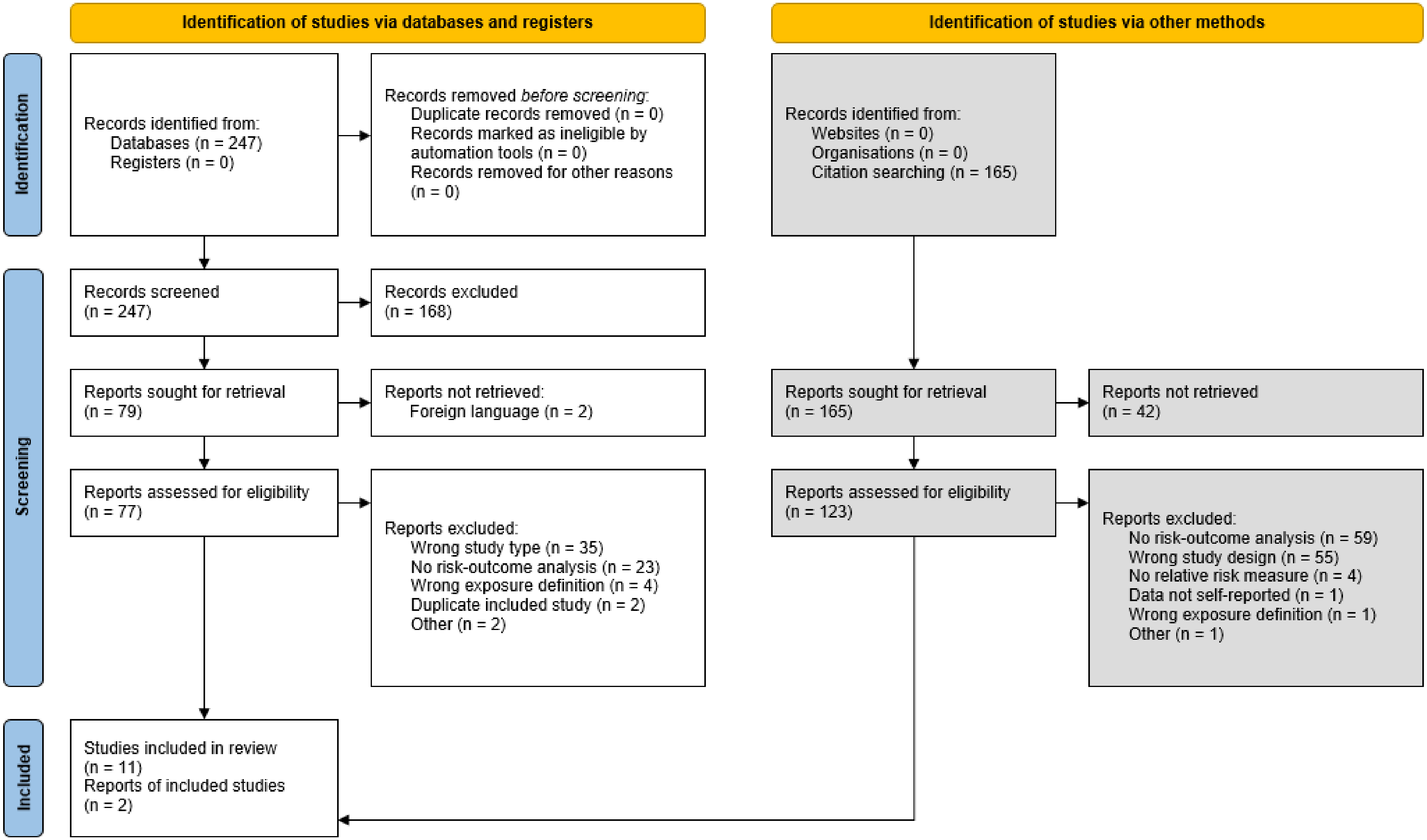


*From:* Page MJ, McKenzie JE, Bossuyt PM, Boutron I, Hoffmann TC, Mulrow CD, et al. The PRISMA 2020 statement: an updated guideline for reporting systematic reviews. BMJ 2021;372:n71. doi: 10.1136/bmj.n71. For more information, visit: http://www.prisma-statement.org/

PRISMA 2020 flow diagram for a new systematic review of the secondhand smoke and breast cancer risk-outcome pair in GBD 2021

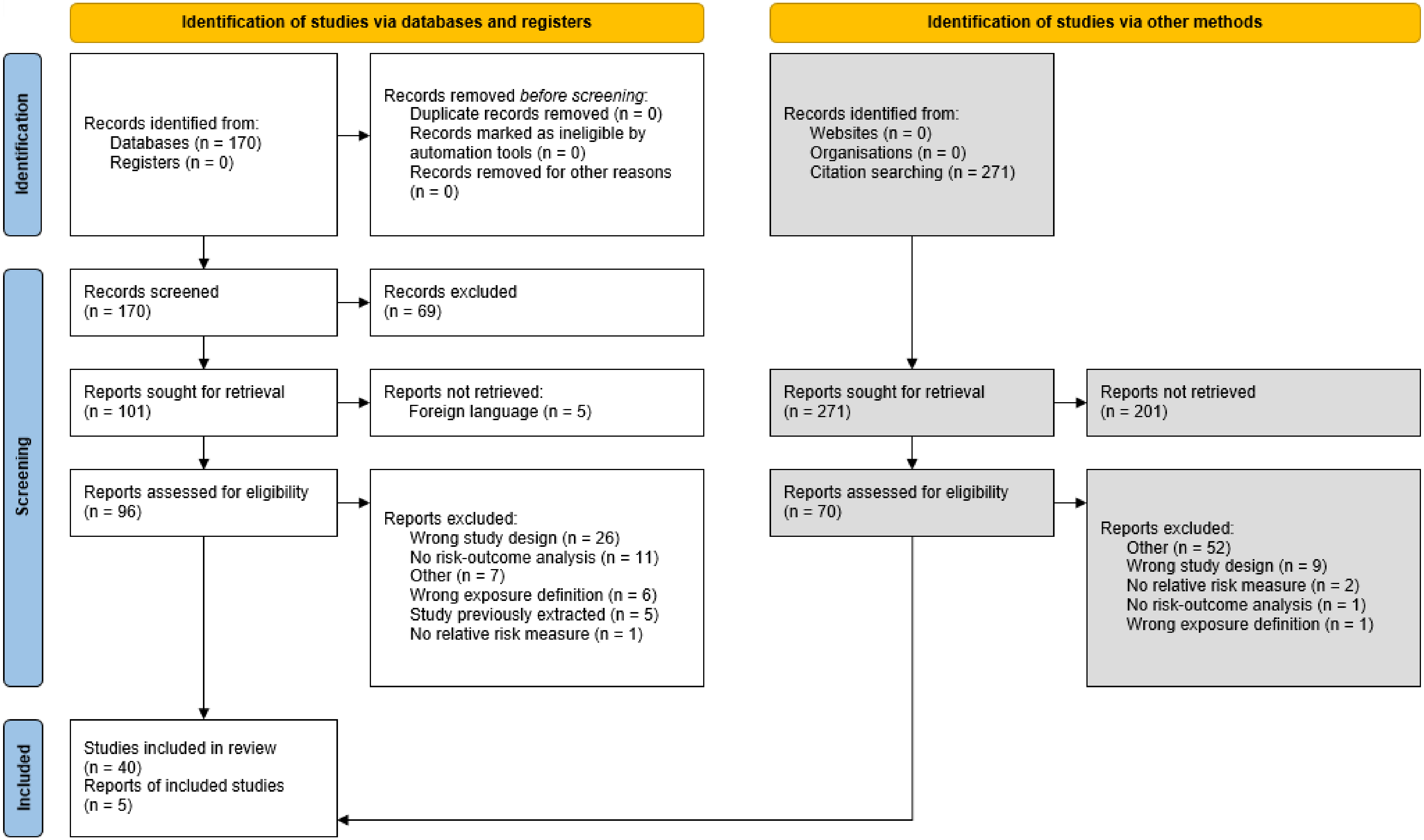

*From:* Page MJ, McKenzie JE, Bossuyt PM, Boutron I, Hoffmann TC, Mulrow CD, et al. The PRISMA 2020 statement: an updated guideline for reporting systematic reviews. BMJ 2021;372:n71. doi: 10.1136/bmj.n71. For more information, visit: http://www.prisma-statement.org/

**PRISMA 2020 flow diagram for a new systematic review of the secondhand smoke and otitis media risk-outcome pair in GBD 2021**

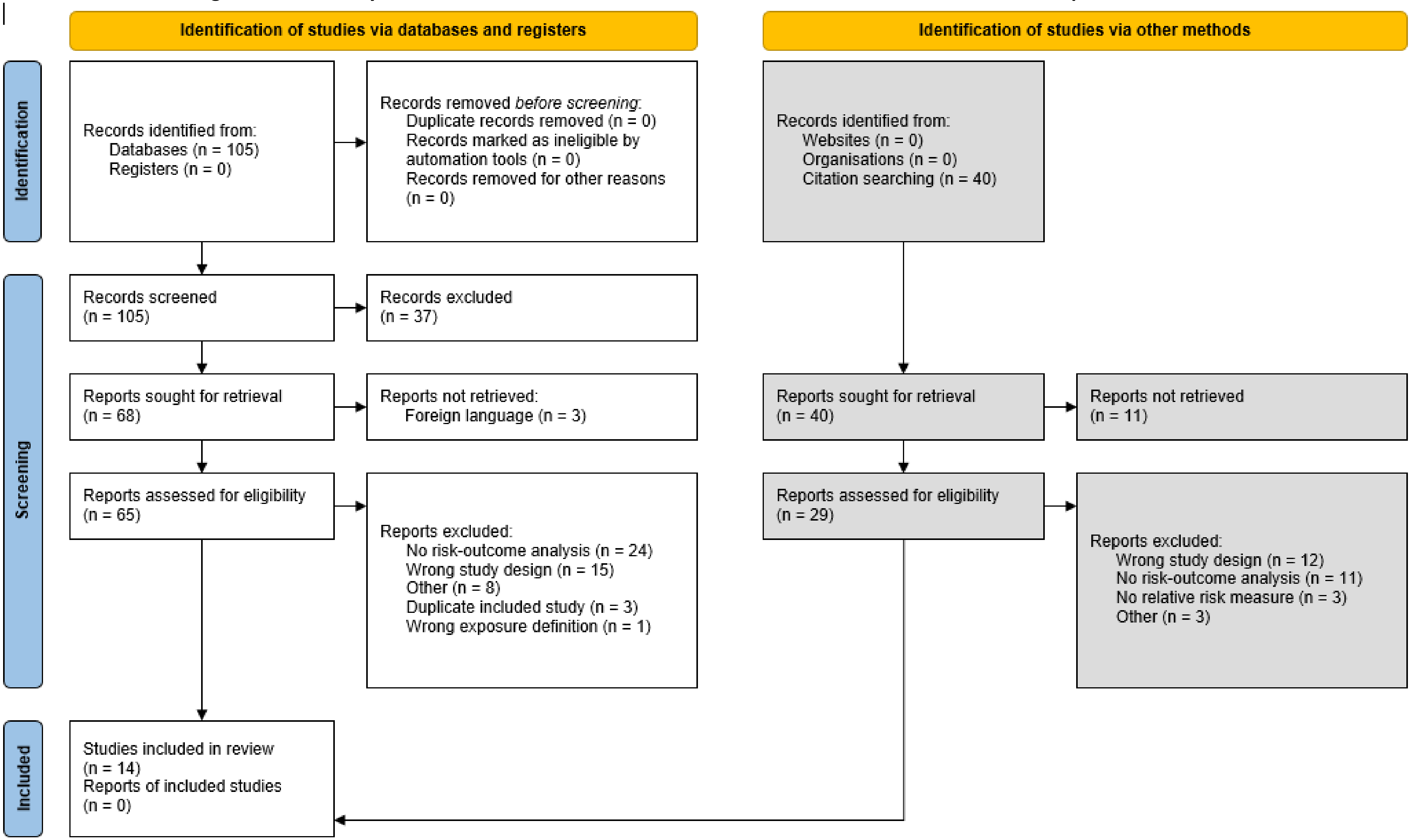


*From:* Page MJ, McKenzie JE, Bossuyt PM, Boutron I, Hoffmann TC, Mulrow CD, et al. The PRISMA 2020 statement: an updated guideline for reporting systematic reviews. BMJ 2021;372:n71. doi: 10.1136/bmj.n71. For more information, visit: http://www.prisma-statement.org/

We included prospective cohort studies and case-control studies that assessed exposure to secondhand smoke as a binary, categorical (level of exposure: low, moderate, high), or continuous (cigarettes per day) exposure, excluding studies that reported exposure using a different continuous metric (eg, number of hours, number of people, number of days, level of cotinine, etc.) or score. Further, we only included studies that reported risk estimates (relative risk, hazard ratio, or odds ratio) with confidence intervals, standard errors, or enough information to quantify uncertainty. In addition, we excluded studies that only reported former exposure to secondhand smoke (eg, child exposure during pregnancy) or only exposure among current smokers. Table 3 summarises the relative risk input data used in GBD 2021.

**Table 3: Data inputs for relative risk for secondhand smoke**

| Input data | Relative risk |
|---|---|
| Source count (total) | 124 |
| Number of countries with data | 33 |

In future rounds of the GBD, we aim to conduct systematic reviews for the outcomes not updated this round and incorporate new evidence for all outcomes as they become available. In addition, we will evaluate the evidence concerning the relationship between exposure to secondhand smoke and other diseases and add these risk-outcome pairs if general GBD inclusion criteria are met.

## Modelling strategy

Prior to GBD 2021, lung cancer, IHD, stroke, and COPD risk curves were calculated jointly with ambient particulate matter pollution, while relative risks for otitis media, breast cancer, and diabetes were derived from published meta-analyses. In GBD 2021, we used the meta-regression—Bayesian, regularised, trimmed (MR-BRT) tool to estimate the log relative risk associated with each level of secondhand smoke exposure on a continuous scale for lung cancer, IHD, stroke, COPD, LRI, and T2DM. For this, we converted binary and categorical exposures reported in each study to a common continuous metric representing the number of cigarettes smoked per smoker per day in each location-year (Table 4). If a study reported exposure in number of cigarettes, we used that number directly.

**Table 4: Converting exposure to a continuous scale**

| Study reported exposure | Matched continuous exposure |
|---|---|
| Binary | Median of the distribution of cigarettes smoked per smoker per day in the study location-year |
| Categorical | **Low:** 25th percentile of the distribution of cigarettes smoked per smoker per day in a specific location-year<br>**Medium:** Median of the distribution of cigarettes smoked per smoker per day in a specific location-year<br>**High:** 75th percentile of the distribution of cigarettes smoked per smoker per day in a specific location-year |
| Continuous (cigarettes per day) | Direct number reported associated with the relative risk reported in the study |

For breast cancer and otitis media, we used the MR-BRT tool to perform our own meta-regression analysis of the risk of developing these conditions for those currently exposed to tobacco smoke relative to the reference category of those not exposed. For these outcomes, only studies reporting a binary exposure were included in the analysis. Table 5 shows the results of the MR-BRT analyses for the outcomes with dichotomous exposure.

**Table 5: Otitis media and breast cancer MR-BRT network meta-analysis results (reference: not exposed to secondhand smoke)**

| Outcome | GBD 2019 relative risk | GBD 2021 MR-BRT relative risk |
|---|---|---|
| Otitis media | 1.37 (1.25–1.50) | 1.23 (1.051.45) |
| Breast cancer | 1.07 (1.02–1.13) | 1.04 (0.951.13) |

For each risk-outcome pair meta-regression, we considered study-level covariates that could potentially bias the study's reported effect size estimates. These study-level covariates included indication of the study design, whether the study used a washout period, whether the study determined outcomes based on administrative records or self-reports, whether the study was generalisable to the general population, and the level of adjustment for relevant confounders like age, sex, smoking, education, and income. We also created covariates to indicate aspects related to the secondhand smoke exposure reported in each study, such as source of exposure (ie, spouse, maternal), exposure setting (ie, work, home, any), exposed population (ie, never smoker, non-smokers), and others. We adjusted for these covariates in our meta-regression if they significantly biased our estimated relative risk function. We used the MR-BRT automated covariate selection process to identify the statistically significant covariates (significance threshold = 0.05). For outcomes with enough datapoints, we introduce likelihood-based trimming to detect and remove outliers (10% trimming) before fitting the model. Outcome-specific model characteristics are described in Table 6.

**Table 6: Risk-outcome pair model specifications and results.**

| Outcome | MR-BRT models specifications | Trimming | Selected covariates | Mean gamma solution | Publication bias |
|---|---|---|---|---|---|
| Continuous (risk curves) | | | | | |
| Ischaemic heart disease | Quadratic splines with 3 internal knots; right linear tail; monotonically increasing constraint; Gaussian prior (0, 0.01) on max derivative of non-linear intervals | Yes | Cohort study | 0.028 | No |
| Ischaemic stroke | Cubic splines with 3 internal knots; right linear tail; monotonically increasing constraint; Gaussian prior | Yes | - | 0.000 | No |

| | | | | | |
|---|---|---|---|---|---|
| | (0, 0.01) on max derivative of non-linear intervals | | | | |
| Chronic obstructive pulmonary disease | Cubic splines with 3 internal knots; right linear tail; monotonically increasing constraint; Gaussian prior (0, 0.01) on max derivative of non-linear intervals | Yes | - | 0.082 | No |
| Lung cancer | Cubic splines with 3 internal knots; right linear tail; monotonically increasing constraint; Gaussian prior (0, 0.01) on max derivative of non-linear intervals | Yes | - | 0.000 | No |
| Lower respiratory infection | Cubic splines with 3 internal knots; right linear tail; monotonically increasing constraint; Gaussian prior (0, 0.01) on max derivative of non-linear intervals | Yes | Adjusted model; Multiple exposure measurements; >95% follow-up | 2.377 | Yes |
| Type 2 diabetes | Cubic splines with 3 internal knots; right linear tail; monotonically increasing constraint; Gaussian prior (0, 0.01) on max derivative of non-linear intervals | No | - | 0.126 | No |
| Dichotomous | | | | | |
| Breast cancer | NA | Yes | Non-smoker population | 0.006 | No |
| Otitis media | NA | Yes | Adjusted model | 0.014 | No |

We implemented the Fisher Scoring correction to the heterogeneity parameter, which corrects for data-sparse situations. In such cases, the between-study heterogeneity parameter estimate may be 0, simply from lack of data. The Fisher Scoring correction uses a quantile of gamma, which is sensitive to the number of studies, study design, and reported uncertainty.

Prior to generating an evidence score, we conducted an additional post-analysis step to test and adjust for publication bias in the input data. This approach is based on the classic Egger's Regression strategy, which is applied to the residuals in our model. In the current implementation, we do not correct for publication bias, but flag the risk-outcome pairs where the risk for publication bias is significant. We found evidence of publication bias for LRI studies only.

There is a well-documented attenuation of the risk for cardiovascular disease throughout one's life. Thus, in GBD 2021, to incorporate this age trend in the relative risks, we first identified the median age-at-event across all IHD and stroke cohorts and considered that as the reference age group. We then assigned our risk curves to this reference age group. Next, we applied 1000 draws of the age-specific attenuation factors produced for the smoking curves to 1000 draws of our reference age group's risk curve to determine age-specific risk curves that propagated the uncertainty of both the risk function and age pattern.

## Population attributable fraction

For outcomes with a risk a curve, we assigned a specific relative risk to each country-year based on the average number of cigarettes smoked per smoker in that location-year. Relative risks for otitis media and breast cancer from MR-BRT were applied to all countries for all years. Except for IHD and stroke, relative risks were applied to all estimated ages. There was no variation in relative risk by sex. We used the standard GBD population attributable fraction equation for dichotomous risks to estimate burden based on exposure, relative risks, and theoretical minimum risk exposure level.
