# Supplementary material for: Global burden of childhood otitis media attributable to secondhand smoke from 1990 to 2021: a systematic analysis of the global burden of disease study 2021
Source: Front Pediatr. 2025 Dec 1;13:1619721. doi: 10.3389/fped.2025.1619721 (PMC12702889; doi:10.3389/fped.2025.1619721)
Supplement: Supplementary file 2 [file Table1.docx]

**Table 1 Global, and regional burden of childhood otitis media due to SHS exposure.**

|  | YLDs | | | | | DALYs | | | | |
| --- | --- | --- | --- | --- | --- | --- | --- | --- | --- | --- |
| location | Number in 1990(95%CI) | Age-standardized rate in 1990 | Number in 2021(95%CI) | Age-standardized rate in 2021 | EAPC | Number in 1990(95%CI) | Age-standardized rate in 1990 | Number in 2021(95%CI) | Age-standardized rate in 2021 | EAPC |
| Global | 95504 (42399,169078) | 5.49 (2.38,9.9) | 78648 (34414,142066) | 3.91 (1.66,7.23) | -1.22 (-1.26,-1.19) | 101472 (46038,178276) | 5.83 (2.61,10.37) | 79560 (34630,143322) | 3.96 (1.68,7.31) | -1.34 (-1.37,-1.3) |
| Sex |  |  |  |  |  |  |  |  |  |  |
| Male | 51248(22832,90850) | 5.74(2.50,10.4) | 43879(19293,80602) | 4.23(1.8,7.82) | -1.13 (-1.17,-1.09) | 54326(24620,94022) | 6.08(2.65,10.81) | 44433(19442,80879) | 4.28(1.82,7.91) | -1.23 (-1.27,-1.19) |
| Female | 44255(19567,78239) | 5.23(2.27,9.44) | 34769(15073,62665) | 3.58(1.51,6.6) | -1.34 (-1.37,-1.31) | 47146(21127,83158) | 4.28(1.82,7.91) | 35127(15457,63019) | 3.62(1.54,6.71) | -1.46 (-1.48,-1.44) |
| SDI |  |  |  |  |  |  |  |  |  |  |
| High SDI | 6916 (3037,12177) | 3.74 (1.61,6.94) | 4449 (1923,8054) | 2.63 (1.11,4.88) | -1.3 (-1.36,-1.24) | 7623 (3470,13114) | 4.14 (1.85,7.35) | 4500 (1952,8114) | 2.66 (1.12,4.92) | -1.5 (-1.59,-1.41) |
| High-middle SDI | 15649 (6942,27797) | 5.73 (2.49,10.37) | 11125 (4901,19908) | 4.83 (2.07,8.97) | -0.69 (-0.74,-0.64) | 16241 (7290,28591) | 5.95 (2.62,10.64) | 11140 (4907,19930) | 4.83 (2.08,8.98) | -0.77 (-0.82,-0.72) |
| Low SDI | 9391 (4057,16610) | 4.11 (1.72,7.46) | 12941 (5573,23659) | 2.81 (1.17,5.23) | -1.39 (-1.46,-1.32) | 11396 (4862,19972) | 4.89 (2.06,8.86) | 13656 (5873,24115) | 2.96 (1.25,5.48) | -1.75 (-1.8,-1.7) |
| Low-middle SDI | 29968 (13210,53216) | 6.35 (2.72,11.37) | 25936 (11392,47954) | 4.48 (1.89,8.34) | -1.17 (-1.2,-1.14) | 31014 (13834,55071) | 6.56 (2.85,11.72) | 26004 (11405,47988) | 4.49 (1.9,8.35) | -1.24 (-1.27,-1.21) |
| Middle SDI | 33509 (15023,59180) | 5.81 (2.55,10.51) | 24138 (10599,43124) | 4.27 (1.83,7.89) | -1.15 (-1.21,-1.1) | 35114 (16159,61416) | 6.08 (2.7,10.89) | 24201 (10633,43231) | 4.28 (1.84,7.91) | -1.25 (-1.32,-1.18) |
| Region |  |  |  |  |  |  |  |  |  |  |
| Andean Latin America | 336 (142,616) | 2.26 (0.95,4.25) | 276 (115,514) | 1.53 (0.62,2.9) | -1.6 (-1.72,-1.49) | 337 (143,616) | 2.27 (0.95,4.26) | 276 (116,515) | 1.53 (0.62,2.9) | -1.61 (-1.72,-1.49) |
| Australasia | 149 (63,268) | 3.28 (1.29,6.33) | 132 (54,249) | 2.36 (0.95,4.56) | -1.16 (-1.2,-1.11) | 154 (65,275) | 3.38 (1.34,6.42) | 132 (54,250) | 2.37 (0.96,4.57) | -1.21 (-1.27,-1.16) |
| Caribbean | 364 (154,662) | 3.2 (1.34,5.95) | 259 (111,485) | 2.26 (0.92,4.27) | -1.46 (-1.59,-1.33) | 366 (155,663) | 3.2 (1.35,5.96) | 260 (111,486) | 2.26 (0.92,4.28) | -1.47 (-1.61,-1.34) |
| Central Asia | 980 (425,1763) | 3.92 (1.68,7.16) | 1033 (455,1846) | 3.72 (1.57,6.83) | -0.16 (-0.25,-0.06) | 987 (429,1772) | 3.94 (1.69,7.2) | 1033 (456,1847) | 3.72 (1.57,6.83) | -0.18 (-0.27,-0.09) |
| Central Europe | 1573 (700,2841) | 5.37 (2.34,9.89) | 754 (330,1373) | 4.3 (1.86,8.02) | -0.78 (-0.81,-0.75) | 2235 (1106,3663) | 7.89 (3.79,13.53) | 761 (335,1385) | 4.35 (1.89,8.09) | -1.59 (-1.83,-1.35) |
| Central Latin America | 2851 (1221,5127) | 4.43 (1.9,8.23) | 1666 (711,3026) | 2.64 (1.11,5.01) | -1.86 (-2.02,-1.7) | 3346 (1523,5787) | 5.19 (2.34,9.2) | 1674 (717,3037) | 2.65 (1.12,5.02) | -2.24 (-2.49,-1.99) |
| Central Sub-Saharan Africa | 718 (302,1295) | 2.84 (1.12,5.43) | 1458 (614,2685) | 2.48 (1.01,4.77) | -0.53 (-0.57,-0.49) | 749 (316,1325) | 2.94 (1.16,5.53) | 1473 (623,2695) | 2.51 (1.02,4.83) | -0.61 (-0.65,-0.57) |
| East Asia | 20183 (9106,36397) | 6.13 (2.72,11.11) | 14164 (6212,25385) | 5.27 (2.23,9.91) | -0.6 (-0.69,-0.5) | 20299 (9171,36528) | 6.16 (2.74,11.16) | 14165 (6213,25387) | 5.27 (2.23,9.91) | -0.61 (-0.71,-0.52) |
| Eastern Europe | 3346 (1473,5921) | 6.49 (2.87,11.81) | 1980 (893,3551) | 5.58 (2.44,10.48) | -0.58 (-0.71,-0.46) | 3357 (1480,5936) | 6.51 (2.88,11.84) | 1981 (893,3552) | 5.58 (2.44,10.48) | -0.59 (-0.71,-0.47) |
| Eastern Sub-Saharan Africa | 2774 (1191,4907) | 3.06 (1.27,5.64) | 4035 (1731,7291) | 2.26 (0.91,4.22) | -1.19 (-1.28,-1.11) | 4733 (2037,8840) | 4.97 (1.96,9.7) | 4786 (2034,8617) | 2.67 (1.08,5.18) | -2.14 (-2.19,-2.09) |
| High-income Asia Pacific | 1391 (613,2464) | 4.02 (1.74,7.58) | 604 (266,1118) | 2.76 (1.14,5.33) | -1.38 (-1.46,-1.3) | 1395 (615,2471) | 4.04 (1.75,7.6) | 604 (266,1118) | 2.76 (1.14,5.33) | -1.39 (-1.46,-1.31) |
| High-income North America | 1904 (831,3398) | 3.09 (1.34,5.7) | 1237 (521,2228) | 1.93 (0.81,3.6) | -1.8 (-1.89,-1.71) | 2028 (919,3581) | 3.29 (1.44,5.93) | 1256 (531,2259) | 1.96 (0.83,3.64) | -1.91 (-2,-1.83) |
| North Africa and Middle East | 6725 (2968,11890) | 9.55 (4.07,17.29) | 7898 (3461,14179) | 8.62 (3.67,15.78) | -0.4 (-0.43,-0.37) | 6726 (2969,11891) | 9.55 (4.08,17.3) | 7898 (3461,14179) | 8.62 (3.67,15.78) | -0.4 (-0.43,-0.37) |
| Oceania | 149 (66,263) | 5.57 (2.42,10.17) | 287 (126,512) | 5.64 (2.42,10.5) | 0.01 (-0.03,0.05) | 150 (67,265) | 5.61 (2.44,10.22) | 288 (126,513) | 5.66 (2.42,10.51) | 0.01 (-0.03,0.05) |
| South Asia | 31444 (13921,55352) | 14.51 (6.23,26.19) | 25067 (11007,47310) | 9.91 (4.14,18.9) | -1.29 (-1.33,-1.25) | 32420 (14303,57817) | 14.95 (6.5,27.05) | 25068 (11008,47311) | 9.91 (4.14,18.9) | -1.36 (-1.4,-1.32) |
| Southeast Asia | 10890 (4870,18832) | 6.38 (2.77,11.4) | 9030 (3951,16104) | 5.25 (2.26,9.67) | -0.69 (-0.74,-0.64) | 10913 (4887,18864) | 6.39 (2.78,11.41) | 9037 (3956,16112) | 5.25 (2.26,9.67) | -0.69 (-0.75,-0.64) |
| Southern Latin America | 636 (275,1151) | 4.27 (1.81,7.93) | 485 (206,885) | 3.43 (1.4,6.55) | -0.84 (-0.89,-0.79) | 645 (280,1164) | 4.33 (1.84,8) | 486 (207,886) | 3.44 (1.41,6.55) | -0.91 (-0.96,-0.87) |
| Southern Sub-Saharan Africa | 1096 (473,1976) | 5.3 (2.25,9.8) | 948 (408,1742) | 3.94 (1.66,7.49) | -1.04 (-1.12,-0.95) | 1130 (493,2011) | 5.46 (2.35,10.03) | 961 (417,1757) | 3.99 (1.7,7.55) | -1.09 (-1.17,-1.01) |
| Tropical Latin America | 3092 (1393,5483) | 5.79 (2.51,10.6) | 1451 (618,2696) | 2.9 (1.22,5.61) | -2.49 (-2.6,-2.38) | 4118 (1991,6790) | 7.84 (3.63,13.49) | 1498 (650,2774) | 2.99 (1.27,5.71) | -3.01 (-3.27,-2.74) |
| Western Europe | 2677 (1167,4870) | 3.83 (1.59,7.31) | 1919 (807,3548) | 2.89 (1.17,5.52) | -0.92 (-0.93,-0.91) | 3158 (1452,5470) | 4.56 (2.03,8.16) | 1954 (820,3602) | 2.95 (1.2,5.58) | -1.29 (-1.38,-1.19) |
| Western Sub-Saharan Africa | 2224 (936,3965) | 2.53 (1.05,4.63) | 3966 (1715,7286) | 1.84 (0.77,3.44) | -1.38 (-1.51,-1.25) | 2225 (937,3966) | 2.53 (1.05,4.63) | 3967 (1715,7287) | 1.84 (0.77,3.44) | -1.38 (-1.51,-1.25) |

SHS: Secondhand smoke.
